# Biochemical and biophysical analyses of hypoxia sensing prolyl hydroxylases from *Dictyostelium discoideum* and *Toxoplasma gondii*

**DOI:** 10.1074/jbc.RA120.013998

**Published:** 2021-01-13

**Authors:** Tongri Liu, Martine I. Abboud, Rasheduzzaman Chowdhury, Anthony Tumber, Adam P. Hardy, Kerstin Lippl, Christopher T. Lohans, Elisabete Pires, James Wickens, Michael A. McDonough, Christopher M. West, Christopher J. Schofield

**Affiliations:** 1 Chemistry Research Laboratory, University of Oxford, Oxford, United Kingdom; 2 Department of Biochemistry and Molecular Biology, Complex Carbohydrate Research Center, Center for Tropical and Emerging Global Diseases, University of Georgia, Athens, Georgia, USA

**Keywords:** hypoxia/oxygen sensor, prolyl-hydroxylase, hypoxia-inducible factor (HIF), 2-oxoglutarate/α-ketoglutarate oxygenase, Dictyostelium discoideum, Toxoplasma gondii, S-phase kinase-associated protein 1 (Skp1), hypoxia, dioxygenase, protein evolution

## Abstract

In animals, the response to chronic hypoxia is mediated by prolyl hydroxylases (PHDs) that regulate the levels of hypoxia-inducible transcription factor α (HIFα). PHD homologues exist in other types of eukaryotes and prokaryotes where they act on non HIF substrates. To gain insight into the factors underlying different PHD substrates and properties, we carried out biochemical and biophysical studies on PHD homologues from the cellular slime mold, *Dictyostelium discoideum,* and the protozoan parasite, *Toxoplasma gondii*, both lacking HIF. The respective prolyl-hydroxylases (DdPhyA and TgPhyA) catalyze prolyl-hydroxylation of S-phase kinase-associated protein 1 (Skp1), a reaction enabling adaptation to different dioxygen availability. Assays with full-length Skp1 substrates reveal substantial differences in the kinetic properties of DdPhyA and TgPhyA, both with respect to each other and compared with human PHD2; consistent with cellular studies, TgPhyA is more active at low dioxygen concentrations than DdPhyA. TgSkp1 is a DdPhyA substrate and DdSkp1 is a TgPhyA substrate. No cross-reactivity was detected between DdPhyA/TgPhyA substrates and human PHD2. The human Skp1 E147P variant is a DdPhyA and TgPhyA substrate, suggesting some retention of ancestral interactions. Crystallographic analysis of DdPhyA enables comparisons with homologues from humans, *Trichoplax adhaerens*, and prokaryotes, informing on differences in mobile elements involved in substrate binding and catalysis. In DdPhyA, two mobile loops that enclose substrates in the PHDs are conserved, but the C-terminal helix of the PHDs is strikingly absent. The combined results support the proposal that PHD homologues have evolved kinetic and structural features suited to their specific sensing roles.

In animals, the chronic response to hypoxia is regulated by prolyl hydroxylases (PHDs), which are Fe(II) and 2-oxoglutarate (2OG)-dependent oxygenases that utilize ferrous iron as a cofactor (1) (Fig. 1). Under normoxic conditions, post-translational prolyl hydroxylation of constitutively produced HIFα targets it for proteasomal degradation via increased affinity for the von Hippel-Lindau protein (pVHL), which is a targeting component of an E3 ubiquitin ligase (2, 3). The HIFs are α,β-heterodimeric transcription factors that up-regulate the expression of multiple genes working in a context-dependent manner to ameliorate the effects of hypoxia at cellular and whole organism levels (4, 5, 6). Asparaginyl hydroxylation of the HIF-α C-terminal transactivation domain (CTAD) is catalyzed by another 2OG oxygenase, factor inhibiting HIF (FIH), which is proposed to fine-tune the expression of specific sets of HIF target genes (3, 7).

**Figure 1:**
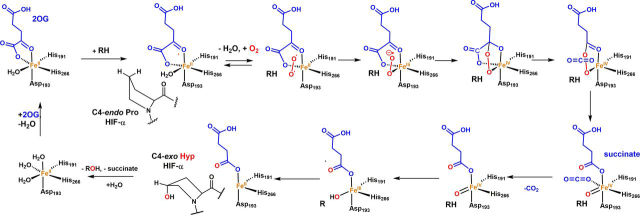
**Outline of the consensus 2OG oxygenase mechanism.** In the resting state, the active site iron is typically coordinated by a triad of residues (HX(D/E)…H motif) and 2-3 water molecules giving octahedral coordination. Sequential binding of 2OG, then substrate, then O_2_ to the active site occurs, causing displacement of the resting state metal-bound waters. Oxidative decarboxylation of 2OG (not all the proposed intermediates have been characterized) gives a ferryl intermediate that is directly responsible for substrate oxidation. In the case of the PHDs the substrate prolyl residue binds in a C4-*endo* conformation but changes to a C4-*exo* conformation on hydroxylation (61, 102).

In humans, there are three PHDs (PHD1-3) and three HIF-α proteins (HIF 1-3α). HIF-1α and HIF-2α each have one asparaginyl-hydroxylation site in their CTAD and two prolyl-hydroxylation sites located in their N- (NODD) and C- (CODD)terminal oxygen-dependent degradation domains (ODDs) (3, 5). HIF-3α has only one prolyl hydroxylation site that is most similar to the HIF-1/2α CODD (8). Of the human PHDs, PHD2 is the most conserved in animals (9). The assignment of the PHDs as hypoxia sensors is supported by cellular, genetic, inhibition, and biochemical studies (10, 11, 12). PHD inhibitors up-regulate erythropoietin levels and have been clinically approved for treatment of anemia in chronic kidney disease patients (13, 14, 15).

Both the PHDs and FIH are structurally related to microbial enzymes involved in antibiotic biosynthesis (16, 17, 18, 19). The PHDs and FIH have distinctive structures and biochemical properties and likely have independent microbial origins, yet both act on HIF in an oxygen availability dependent manner, in an apparent example of convergent evolution to a common function. FIH belongs to the JmjC structural family VI of 2OG oxygenases (11, 20), which includes the ribosomal oxygenases present in eukaryotes and prokaryotes (Mina53/NO66 and YcfD, respectively) and the N^ε^-methyl lysyl/arginyl demethylases (KDMs) (17, 18, 19, 21, 22, 23). The PHDs belong to the prolyl hydroxylase (PH) structural family VIII of 2OG oxygenases, which includes the procollagen/collagen and ribosomal protein prolyl hydroylases (16, 20).

The conserved hypoxia sensing role of the HIF-PHD-pVHL triad in humans and other animals raised questions as to its evolutionary origins and whether other 2OG oxygenase-mediated hypoxia-sensing mechanisms exist (2, 24). In animals, for example, some of the JmjC KDMs are proposed to be involved in the hypoxic response (25, 26, 27).

Multiple nonHIF-α substrates from different protein families have been reported for the human PHDs; however, a recent study using isolated PHD enzymes reports conflicting results, *i.e.* all the tested nonHIF-α substrates tested were found not to be hydroxylated, although it is possible that substrate specificity is different in cells (28).

Experimental and bioinformatic studies on the evolution and extent of the HIF-PHD-pVHL system across biology (3, 29, 30, 31) have revealed PHD-type enzymes in a range of nonmetazoan-type eukaryotes (9, 32) and some bacteria (33). Studies on a PHD from the simple animal *Trichoplax adhaerens* (TaPHD), have led to validation of a functional HIF system in this organism (9, 34). A PHD-like enzyme from *Pseudomonas putida* (PPHD) has been shown to catalyze prolyl hydroxylation of the ubiquitous protein elongation factor-thermally unstable (EF-Tu), implying a role for prolyl hydroxylation in regulation of the translation machinery in bacteria (33, 35).

In the cellular slime mold *Dictyostelium discoideum*, the human parasite *Toxoplasma gondii* and the crop pathogen *Pythium ultimum*, PHD-type 2OG oxygenases catalyze the hydroxylation of a prolyl residue in the S-phase kinase-associated protein (Skp1) (36, 37) (Fig. 2, *A*, *D*, and *E*). This enables Skp1 glycosylation in a manner proposed to regulate protein degradation via effects on the SCF class of E3-ubiquitin ligase of which Skp1 is an essential subunit (33, 38); however, glycosylation independent roles of Skp1 prolyl hydroxylation are also manifested (39, 40, 41, 42, 43, 44, 45). In humans, the Skp1 prolyl residue that undergoes hydroxylation (Pro-143 in Skp1 from *D. discoideum* and Pro-154 in Skp1 from *Tg*) is substituted for a glutamate (Glu-147 in human Skp1) (46). In *Toxoplasma gondii*, a prolyl hydroxylase (TgPhyB) regulates translation elongation, potentially through oxygen dependent hydroxylation (47).

**Figure 2:**
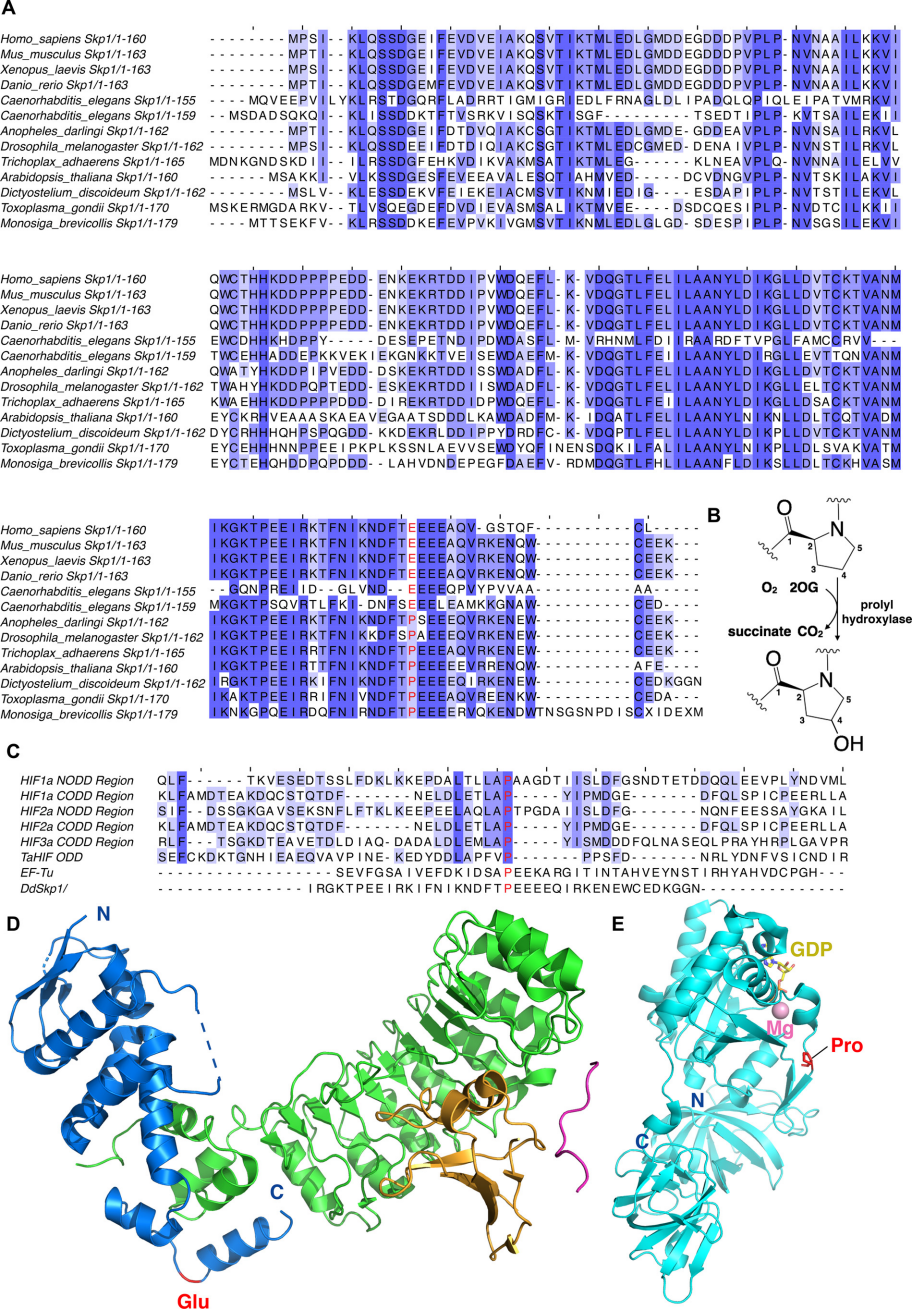
**Sequence alignment of eukaryotic Skp1 proteins.***A,* Skp1 sequences are from the following organisms: human (*Homo sapiens*, Gene ID NP_008861.2), mouse (*Mus musculus*, Gene ID NP_035673.3), frog (*Xenopus laevis*, Gene ID AAF65619.1), zebra fish (*Danio rerio*, Gene ID NP_957037.1), roundworm (*Caenorhabditis elegans*, Gene ID NP_510193.4), roundworm (*Caenorhabditis elegans*, Gene ID NP_507857.1), mosquito (*Anopheles darlingi*, Gene ID ETN58923.1), fruit fly (*Drosophila melanogaster*, Gene ID NP_477390.1), *T. adhaerens* (Gene ID EDV18896.1), *Arabidopsis thaliana* (Gene ID AEE35780.1), *D. discoideum* (Gene ID XP_644826.1), *T. gondii* (Gene ID CAJ20499.1), and *Monosiga brevicollis* (Gene ID EDQ90844.1, predicted protein). The (potentially) hydroxylated prolyl residue, as occurs for PhyA in *D. discoideum* and *T. gondii*, is in *red*. Residue 147 (HsSkp1, for example) is a glutamyl residue in many complex animals. *B,* prolyl hydroxylases catalyze prolyl-4-hydroxylation. *C,* sequence alignment of prolyl hydroxylase substrates. Human HIF-1α (Gene ID AAC68568.1), human HIF-2α (Gene ID Q99814.3), human HIF-3α (Gene ID Q9Y2N7.2), *T. adhaerens* HIF (Gene ID AFM37575.1), and *P. putida* EF-Tu (Gene ID B1JDW6.1). The (potentially) hydroxylated prolyl residue is in *red*. *D,* view from a structure of human Skp1 in the Skp1-Skp2-Cks1-p27 peptide complex (PDB code 2AST) (103). Skp1 is shown as a *blue* cartoon, Skp2 is in *green*, Cks1 is in *orange*, and the p27 peptide is in *pink*. The glutamyl residue is in *red*. *E,* view from a structure of *E. coli* EF-Tu (PDB code 5MI3) (104). EF-Tu is shown as a *light blue* cartoon. The hydroxylated prolyl residue by the PHD is in *red sticks*; Mg^2+^ is *pink*, and GDP is *yellow*.

Crystal structures of human PHD2 (HsPHD2), PPHD, and TaPHD reveal a distorted double-stranded β-helix (DSBH) core-fold (strands βI-βVIII) present in other 2OG oxygenases, which supports highly conserved Fe(II) and 2OG-binding residues (18, 19, 20, 21). The combined biophysical (X-ray and NMR) analyses reveal conserved mobile elements involved in induced fit-type substrate binding. In both TaPHD and HsPHD2, these include active site bordering loops, *i.e.* a “finger loop” linking β2 and β3 in HsPHD2, and a βIV-βV “insert loop” (Roman numerals refer to the 8 core DSBH strands) and an often α-helix containing C-terminal region (18, 19, 20, 21). The structures also reveal clear differences in aspects of substrate binding by the animal PHDs and bacterial PPHD (33, 48). These in part reflect substantial conformational changes that are required to enable catalytically productive binding of the well-structured EF-Tu to PPHD (33). By contrast, the HIF-α NODD/CODD regions that are PHD substrates are substantially disordered (49), although portions of them adopt defined secondary structure when bound to the PHDs (16, 34, 50).

A detailed understanding of how the PHDs and related prolyl hydroxylases bind their substrates is of interest from an evolutionary perspective may help in predicting their substrates and in designing selective inhibitors both for the human PHDs and PHD-like enzymes in human pathogens. Such an understanding has been hindered by a dearth of structures of PHD-type oxygenases that accept nonHIF-α substrates. Aside from studies on human PHD and TaPHD (16, 34, 50), both of which have HIF-α substrates, there are no reports of structures of eukaryotic PHD homologs, nor on the cross-reactivity of prolyl hydroxylases with the (potential) substrates reported from different types of eukaryotes. Here we report biochemical and biophysical studies on the PHD-like enzymes from *D. discoideum* (DdPhyA) and *T. gondii* (TgPhyA). Because peptide fragments of their Skp1 substrates were inefficiently hydroxylated, we developed assays with intact protein substrates. Combined with crystallographic studies on DdPhyA, the solution studies inform on similarities and differences in PHD-type enzymes and on how the HIFα/Skp1 prolyl hydroxylases achieve substrate specificity.

## Results

### Hydroxylation of DdSkp1 and TgSkp1 by DdPhyA and TgPhyA

**Figure 3:**
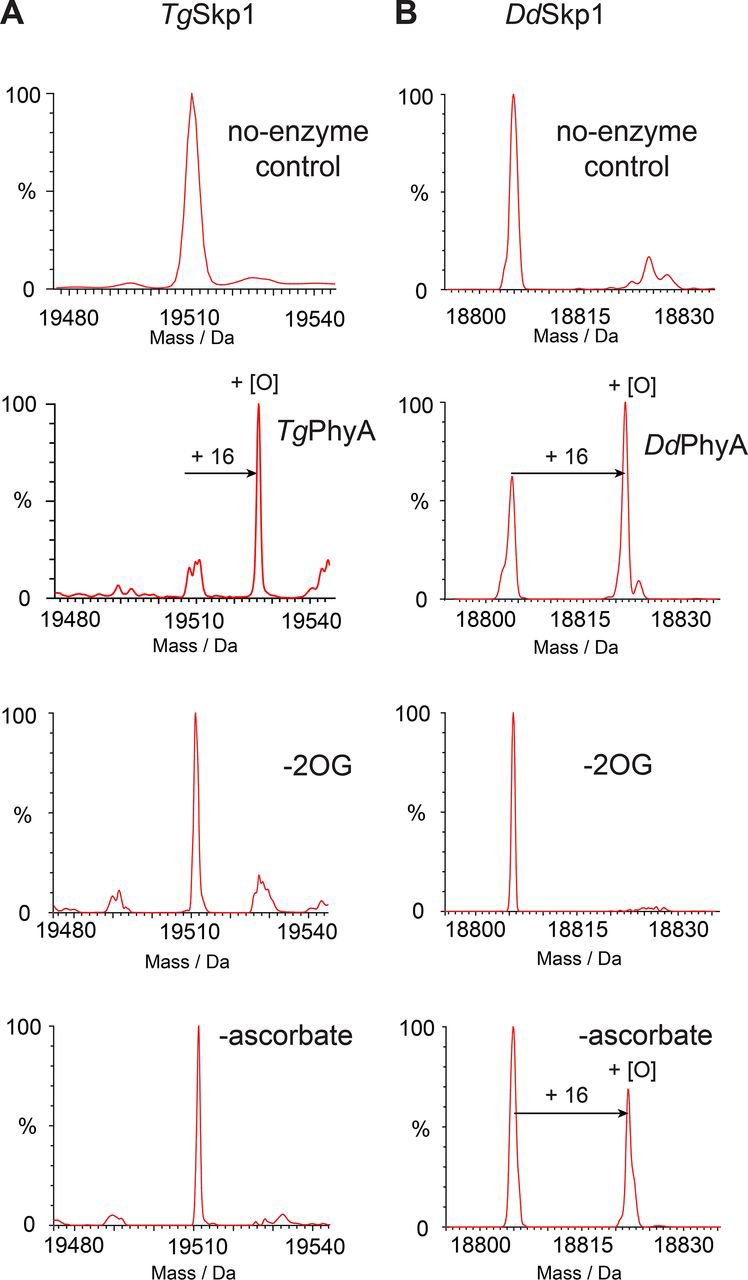
**Mass spectra of full-length DdSkp1 and TgSkp1 proteins after incubation under varied conditions (+16 Da peak shift indicates DdPhyA and TgPhyA hydroxylation activity).***A,* TgSkp1 hydroxylation requires TgPhyA, 2OG, and is enhanced by ascorbate. *B,* DdSkp1 is hydroxylated by DdPhyA in the presence of 2OG; l-ascorbate increases DdPhyA activity, but to a lesser extent than TgPhyA. Assays were performed in the presence or absence (no-enzyme control top panels) of DdPhyA or TgPhyA (1 μm), full-length DdSkp1 or full-length TgSkp1 (100 μm), (NH_4_)_2_Fe(II)(SO_4_)_2_ (50 μm), sodium l-ascorbate (1 mm), and 2OG (500 μm) in HEPES (100 mm), pH 7.6. Reactions were incubated at 37 °C for 1 h and quenched using an equal volume of 1% (v/v) aqueous formic acid, then subjected to LC-ESI-MS analysis. Assay details are provided under the Supporting information.

To investigate use of intact protein substrates for DdPhyA and TgPhyA, recombinant forms of full-length DdPhyA and TgPhyA, along with the *D. discoideum*, *T. gondii,* and human Skp1 substrates (DdSkp1, TgSkp1, HsSkp1, and HsSkp1(E147P)) were produced to >90% purity as judged by SDS-PAGE and electrospray ionization MS (ESI-MS) analyses. Purified DdPhyA and TgPhyA were found to catalyze hydroxylation of their WT substrates, *i.e.* full-length DdSkp1 and TgSkp1 proteins, respectively, in a 2OG-dependent manner as evidenced by a +16 Da mass shift using ESI-MS (Fig. 3). Catalytic activity was enhanced by the addition of l-ascorbate, with the activity of TgPhyA being strongly promoted by l-ascorbate, *i.e.* <10% turnover was observed in its absence (Fig. 3).

Fragmentation MS was used to identify the hydroxylation sites of the full-length protein substrates. Consistent with prior assignments, the results reveal that DdPhyA and TgPhyA only catalyze hydroxylation of Pro^143^ in DdSkp1 and Pro^154^ in TgSkp1, respectively (Figs. 2*A* and Figs. S3 and S4) (51, 82). There was no evidence for hydroxylation/other hydroxylase-catalyzed modification of other prolyl residues in the Skp1 substrates, consistent with the lack of reported evidence for post-translational modifications of DdSkp1/TgSkp1 other than for prolyl hydroxylation/glycosylation.

We investigated the regiochemistry of DdSkp1 and TgSkp1 prolyl hydroxylation by NMR spectroscopy using Skp1 fragment peptide substrates (Fig. S5). Consistent with previous reports, the results confirmed that DdPhyA and TgPhyA catalyze C-4 prolyl hydroxylation, as shown for some, but not all, other prolyl hydroxylases (33, 34, 52, 53).

### Substrate selectivities of DdPhyA and TgPhyA

**Figure 4:**
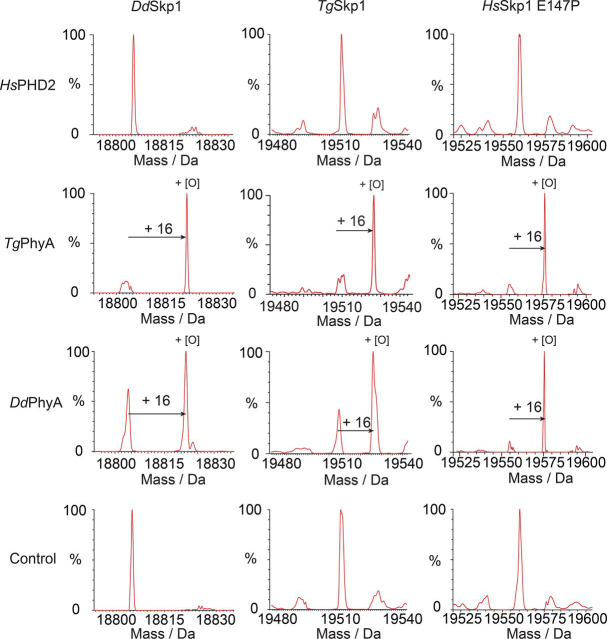
**Substrate selectivity comparison of DdPhyA, TgPhyA, and HsPHD2.***Rows* are specified by enzyme type or control; *column headers* indicate the substrate used. LC-ESI-MS analysis reveals HsPHD2 does not catalyze hydroxylation of any of the Skp1 proteins (DdSkp1, TgSkp1, or HsSkp1(E147P)). Hydroxylation of full-length DdSkp1 protein by DdPhyA and TgPhyA occurs in the presence of 2OG and ascorbate. Full-length TgSkp1 is hydroxylated by both DdPhyA and TgPhyA under the same conditions. Full-length HsSkp1(E147P) is hydroxylated by DdPhyA and TgPhyA. Assays were performed under atmospheric O_2_ concentrations and contained HsPHD2, DdPhyA, or TgPhyA (1 μm), either full-length HsSkp1(E147P), full-length DdSkp1 or full-length TgSkp1 substrate (100 μm), (NH_4_)_2_Fe(II)(SO_4_)_2_ (50 μm), sodium l-ascorbate (1 mm), and 2OG (500 μm) in HEPES (100 mm), pH 7.6. Reactions were incubated at 37 °C (1 h), then quenched with 1% (v/v) aqueous formic acid and subjected to LC-ESI-MS analysis. Assay details are provided under the Supporting information.

We then investigated the substrate selectivity of DdPhyA and TgPhyA and compared the results with HsPHD2. The results reveal, at least under the conditions tested, that the HIF-α ODDs were the only substrates hydroxylated by HsPHD2 (Table 1, Table 2), *i.e.* HsPHD2 did not hydroxylate any of the Skp1 substrates tested, consistent with a recent study reporting that isolated PHDs are highly selective for HIF-α. By contrast, the results indicate that all the Pro^143^ (or equivalent) containing Skp1 proteins tested, *i.e.* DdSkp1 and TgSkp1, are substrates for both DdPhyA and TgPhyA (Fig. 4, Table 1, Table 2). Notably, although none of the enzymes catalyzed hydroxylation of HsSkp1, consistent with its lack of an appropriately positioned prolyl residue (it has a glutamyl residue at this position), both DdPhyA and TgPhyA catalyzed hydroxylation of HsSkp1 in which its Glu^147^ was substituted for a prolyl residue (HsSkp1(E147P)), consistent with previous findings for DdPhyA using an indirect assay (53).

**Table 1:** Summary of PHD-related selectivity results Ticks and crosses indicate hydroxylation/no-hydroxylation under standard conditions. All peptide and protein details are provided under the supporting data. Bottom, comparison of *K_m_* values for 2OG oxygenases involved in hypoxia sensing using MS based assays. Data for HsPHD2(181-426) catalyzed hydroxylation of HIF-α C- and N-terminal oxygen-dependent degradation domains (CODD and NODD, respectively) and FIH catalyzed hydroxylation of the HIF-α CTAD are from Tarhonskaya *et al*. (54, 56). Peptide sequences are given in Table S1.

	DdPhyA	TgPhyA	HsPHD2
DdSkp1 (full-length protein)	✓	✓	✗
TgSkp1 (full-length protein)	✓	✓	✗
HsSkp1 (full-length protein)	✗	✗	✗
HsSkp1 E147P (full-length protein)	✓	✓	✗
Hs HIF1α-CODD (peptide)	✗	✗	✓ (34)
Hs HIF1α -NODD (peptide)	✗	✗	✓ (34)

**Table 2:** Summary of PHD-related selectivity results Comparison of *K_m_* values for 2OG oxygenases involved in hypoxia sensing using MS based assays. Data for HsPHD2(181–426) catalyzed hydroxylation of HIF-α C- and N-terminal oxygen-dependent degradation domains (CODD and NODD, respectively) and FIH catalyzed hydroxylation of the HIF-α CTAD are from Tarhonskaya *et al*. (54, 56). Peptide sequences are given in Table S1.

Enzyme	Substrate	$K_{m}^{\text{app}}$ (substrate)	$K_{m}^{\text{app}}$ (O_2_)	$K_{m}^{\text{app}}$ (2OG)
		μ*m*		
FIH (54)	HIF-1α CTAD	180 ± 30	180 ± 30	110 ± 20
FIH (54)	HIF-2α CTAD	315 ± 40	110 ± 10	19 ± 6
PHD2 (54)	HIF-1α	10 ± 6	460 ± 30	13 ± 2
PHD2 (54)	HIF-1α NODD	11 ± 2	>450	30 ± 9
PHD2 (54)	HIF-2α NODD	34 ± 10	>450	9 ± 2
PHD2 (54)	HIF-2α NODD	50 ± 8	410 ± 80	17 ± 5
DdPhyA	DdSkp1 protein	350 ± 77	>60%	36 ± 6
TgPhyA	TgSkp1 protein	202 ± 47	(0.2 ± 0.04)%

### Kinetics of DdPhyA and TgPhyA using full-length protein substrates

**Figure 5:**
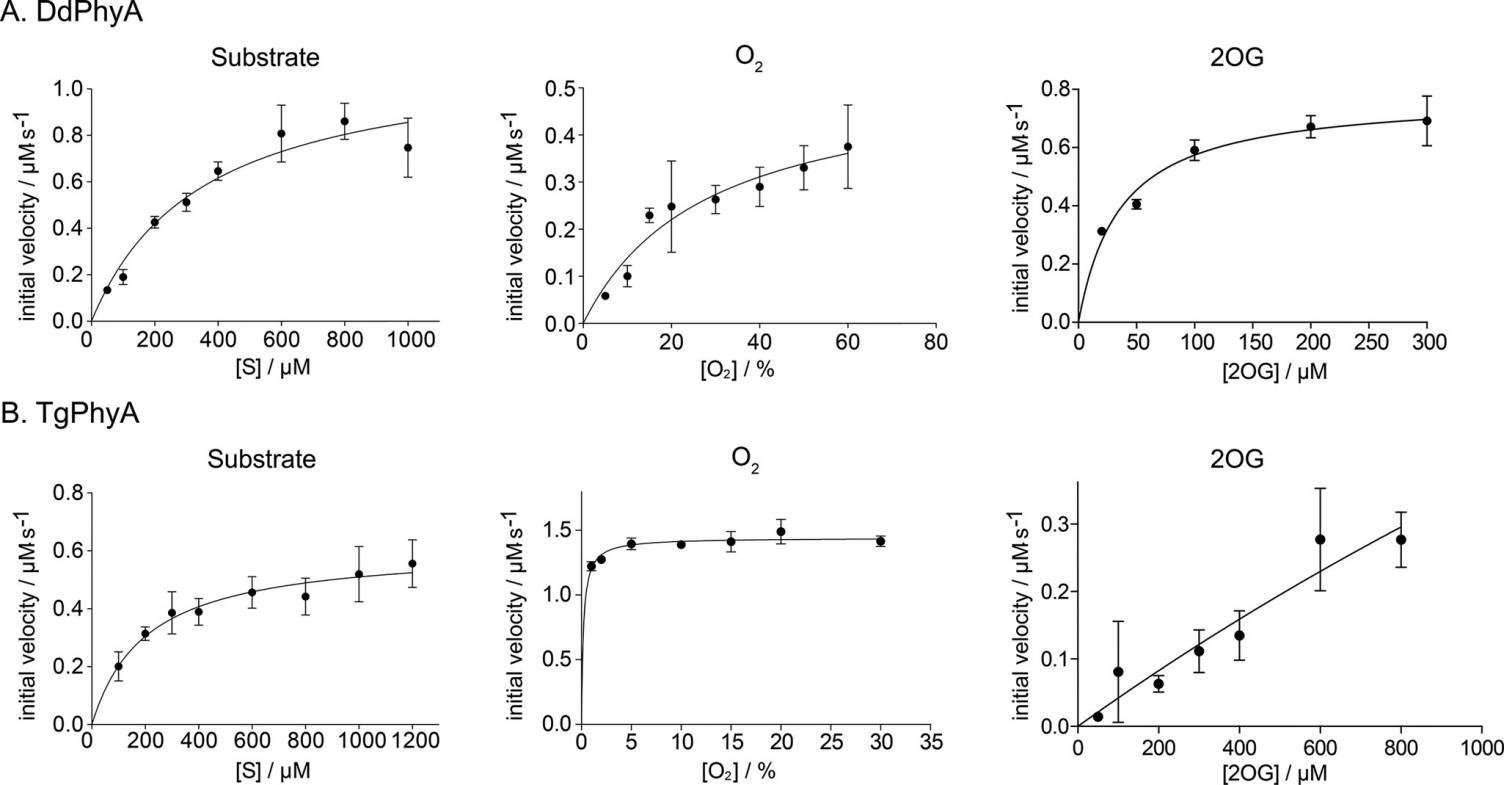
**Kinetics of DdPhyA and TgPhyA.***A,* studies on the dependence of DdPhyA activity on concentrations of: substrate (full-length DdSkp1) (500 μm 2OG, 50 μm (NH_4_)_2_Fe(II)(SO_4_)_2_, 1 mm l-ascorbate, normoxic, 4 min, Xevo LC-ESI-MS); 2OG (DdPhyA (1 μm), full-length DdSkp1 substrate (100 μm), (NH_4_)_2_Fe(II)(SO_4_)_2_ (50 μm), sodium l-ascorbate (1 mm), normoxic, 6 min, RapidFire MS); and O_2_ (DdPhyA (1 μm), full-length DdSkp1 substrate (600 μm), (NH_4_)_2_Fe(II)(SO_4_)_2_ (50 μm), sodium l-ascorbate (1 mm) and 2OG disodium salt (500 μm), 8.25 min, Xevo LC-ESI-MS). All assays were in HEPES (100 mm), pH 7.6, at 37 °C. Assays using Xevo LC-ESI-MS were in triplicate; those using RapidFire MS were in duplicate. *B,* studies on the dependence of TgPhyA activity on concentrations of: substrate (full-length TgSkp1) (500 μm 2OG, 50 μm (NH_4_)_2_Fe(II)(SO_4_)_2_, 1 mm l-ascorbate, normoxic, 4 min, Xevo LC-ESI-MS); 2OG (TgPhyA (1 μm), full-length TgSkp1 substrate (600 μm), (NH_4_)_2_Fe(II)(SO_4_)_2_ (50 μm), sodium l-ascorbate (1 mm), normoxic, 6 min, RapidFire MS); and O_2_ (TgPhyA (1 μm), full-length TgSkp1 substrate (600 μm), (NH_4_)_2_Fe(II)(SO_4_)_2_ (50 μm), sodium l-ascorbate (1 mm) and 2OG disodium salt (500 μm), 8.25 min, Xevo LC-ESI-MS). All assays were in HEPES (100 mm), pH 7.6, at 37 °C. Assays using Xevo LC-ESI-MS were in triplicate; those using RapidFire MS were in duplicate. Note the high level of TgPhyA uncoupled 2OG turnover complicates 2OG $K_{m}^{\text{app}}$ determination, see text. Assay details are provided in the Supporting information.

We then analyzed the kinetics of DdPhyA and TgPhyA catalysis using full-length protein substrates with monitoring for hydroxylation by LC ESI-MS (Fig. 5). It should be noted that most prior kinetic analyses on 2OG oxygenases acting on proteins, including much of the work on the human PHDs, have been carried out with peptide fragments of the validated protein substrates (2, 9, 28, 33, 34, 51, 54, 55). Notably, even with the full-length protein substrates the $K_{m}^{\text{app}}$ values for the substrates of DdPhyA and TgPhyA were relatively high (*i.e.* 350 ± 77 μm for DdPhyA and 202 ± 47 μm for TgPhyA) compared with the values reported for HIF-α ODD PHD substrates, although they are comparable with those reported for FIH HIF-α CTAD hydroxylation (54). The DdPhyA $K_{m}^{\text{app}}$ for 2OG (∼35 μm) was in the same range as reported for other prolyl hydroxylases (56). However, due to a lack of saturation it was not possible to measure an accurate value for the TgPhyA 2OG *K_m_*; the reason for this observation is unclear, but may be related to high level of “uncoupled” 2OG turnover in the absence of substrate for TgPhyA (see below).

There was also a clear difference in the sensitivities of DdPhyA and TgPhyA to variation in dioxygen concentrations as measured by intact protein hydroxylation assays. The estimated DdPhyA $K_{m}^{\text{app}}$ for O_2_ was notably high (>60% atmospheric O_2_), whereas that for TgPhyA was substantially lower (<0.2%), in support of previous reports (57, 82). The possibility of substrate-uncoupled 2OG turnover, in particular for TgPhyA (see below), should be considered in comparing these results, although since this consumes oxygen, this should not alter the conclusion that TgPhyA is more active than DdPhyA at low oxygen concentrations.

The different sensitivities of isolated DdPhyA and TgPhyA to varied O_2_ concentrations are consistent with the results of biological studies on DdPhyA and TgPhyA. Thus, only partial DdSkp1 hydroxylation is observed in *D. discoideum* cells at 5% O_2_ (44, 59). By contrast, biological studies on TgPhyA show efficient TgSkp1 hydroxylation in cells unless O_2_ availability is very low (32, 83).

### 2OG turnover by DdPhyA, TgPhyA, and HsPHD2

We used NMR spectroscopy to monitor 2OG turnover to succinate in real time by DdPhyA, TgPhyA, and, for comparison, HsPHD2 (Figs. S6, S7, and Table S2), in the presence and absence of various substrates. Without substrate, 2OG turnover by DdPhyA was low (<10% in 1 h); however, 2OG turnover was strongly stimulated by the addition of all the Skp1 substrates, *i.e.* TgSkp1, HsSkp1(E147P), and, most strongly, by DdSkp1. Interestingly, with HsSkp1, which is not a DdPhyA substrate, 2OG turnover by DdPhyA was completely inhibited under our conditions suggesting that HsSkp1 might compete with 2OG binding, potentially by a mechanism involving HsSkp1(E147P) side chain coordination of the DdPhyA active site metal (Fig. S6A). Similarly, with TgPhyA, HsSkp1 significantly inhibited 2OG turnover in the absence of substrate, whereas DdSkp1 and, more strongly, TgSkp1 stimulated 2OG turnover. Notably, HsSkp1(E147P) induced the most efficient 2OG turnover with TgPhyA (Fig. S6B). As reported (60, 61), HsPHD2 catalyzed little 2OG turnover (<10% in 1 h) in the presence (or absence) of any of the Skp1 proteins (Fig. S6C). By contrast with HsPHD2 and DdPhyA, TgPhyA exhibited substantial uncoupled 2OG turnover in the absence of substrate, with >80% 2OG turnover being observed by NMR within 1 h, in the presence of l-ascorbate (Fig. S6D). In the absence of l-ascorbate, uncoupled 2OG turnover by TgPhyA was greatly reduced (<5%) (Fig. S6E). Skp1 substrates did not promote 2OG turnover in the absence of enzyme. No binding of 2OG to Skp1 was observed by Carr-Purcell-Meiboom-Gill (CPMG)-edited ^1^H NMR spectroscopy, as indicated by the lack of peak intensity reduction or broadening of the 2OG methylene resonance at 2.22 ppm (Fig. S8).

The combined biochemical analyses reveal substantial differences in the kinetics of DdPhyA and TgPhyA, in particular with respect to variations in their O_2_ dependence and the extent of 2OG turnover in the absence of substrate. Although both DdPhyA and TgPhyA catalyzed hydroxylation of all the tested Skp1s with appropriately positioned prolyl residues, the distinctive results for the two enzymes suggests differences in the way they bind and react with O_2_, 2OG, and their substrates.

### Structural analysis of DdPhyA(60-284)

To investigate the structural basis of the differences between DdPhyA, TgPhyA, and other PHD/PHD-like oxygenases, we worked to obtain crystal structures of them. Following construct optimization and use of thermal shift assays, which revealed that Ni(II) ions and *N*-oxalylglycine (a stable 2OG analog, Fig. S9) stabilize the enzymes, we obtained crystals of DdPhyA(60-284) (space group *C*222_1_, 3 molecules per asymmetric unit, resolution 2.02 Å, Table 3) from which we were able to determine a structure by molecular replacement.

**Table 3:** Crystallographic data processing and refinement statistics for the DdPhyA structure (PDB code 6T8M)

**Data collection**	
Beamline (wavelength, Å)	Diamond light source IO3 (0.97623)
Detector	PILATUS 6M-F
Data processing	XDS
Space group	C 2 2 21
Cell dimensions *a*, *b*, *c* (Å)	45.21, 81.74, 379.07
No. of molecules/ASU	3
Resolution (Å)	47.38–2.02 (2.13–2.02)^a^
No. of unique reflections	47,025 (6792)^a^
Completeness (%)	100.0 (100.0)^a^
Redundancy	9.1 (9.1)^a^
*R*_sym_^b^	0.103 (1.426)^a^
Mean *I*/σ(*I*)	12.2 (1.7)^a^
**Refinement**	
*R*_factor_^c^	0.216
*R*_free_^c^	0.256
Root mean square deviation	
Bond length (Å)	0.004
Bond angle (°)	0.685
Ramachandran plot	
Most favored region	96.59
Additionally allowed region	3.41
Disallowed region	0.0


a Highest resolution shell is shown in parentheses.

b *R*_sym_ = Σ| *I*−〈*I*〉 |/Σ*I,* where *I* is the intensity of an individual measurement and 〈*I*〉 is the average intensity from multiple observations.

c *R*_factor_ = Σ*_hkl_*| |*F*_obs_(*hkl*)| − |*F*_calc_(*_hkl_*)| |/Σ|*F*_obs_(*hkl*)| for the working set of reflections; *R*_free_ is the *R*_factor_ calculated for ∼5% of reflections that were excluded from refinement.

**Figure 6:**
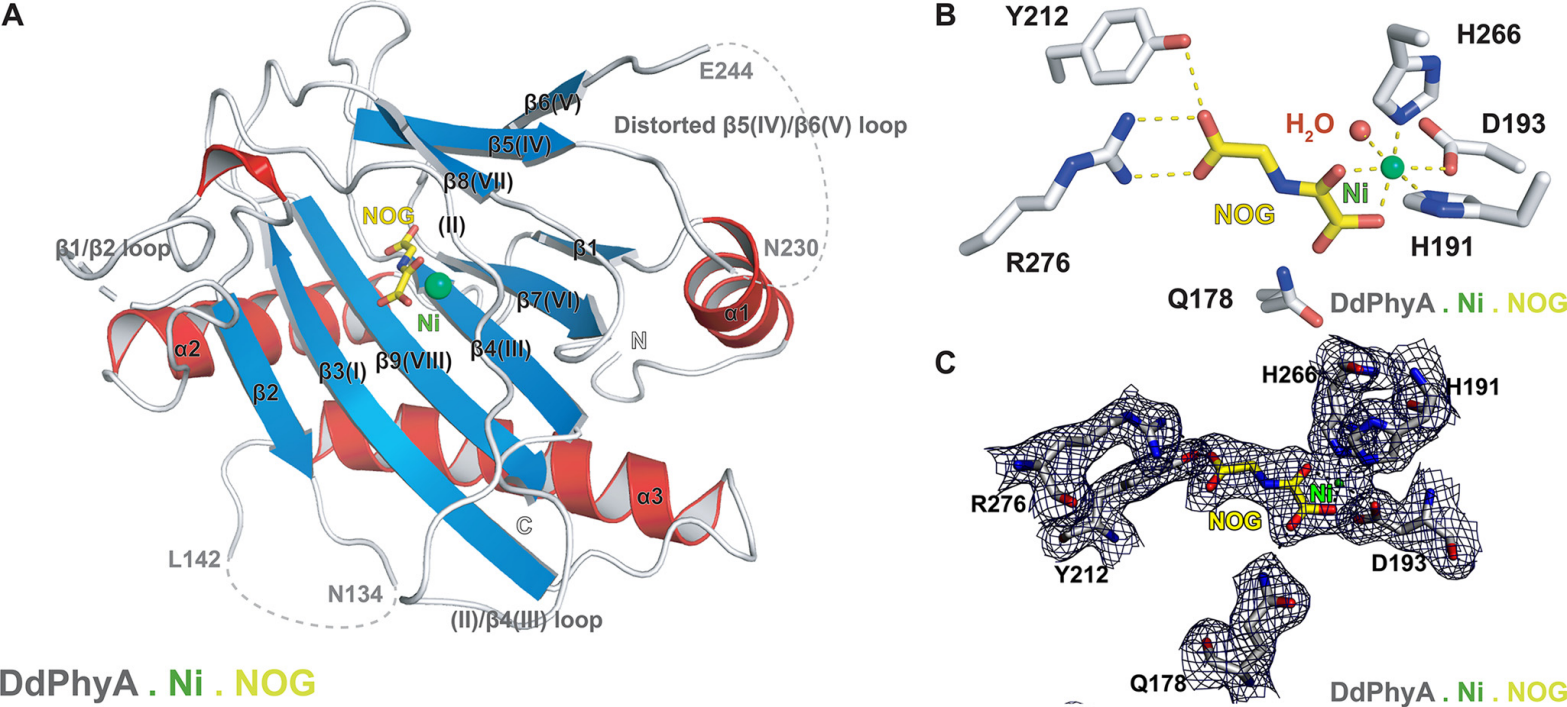
**Views from a crystal structure of the *D. discoideum* prolyl hydroxylase (DdPhyA(60-284)) (PDB code**6T8M**).***A,* secondary structural elements in DdPhyA comprise 3 α-helices and 9 β-strands, 7 of which, together with one loop (II), form the DSBH core-fold (I–VIII). *B,* active site close-up; the metal ion (nickel substituting for iron, *green sphere*) is octahedrally coordinated by His^266^, Asp^193^, and His^191^, NOG (a close 2OG analog), and a water (*red sphere*). NOG is positioned to interact with Arg^276^ via a salt bridge, *i.e.* between the NOG carboxyl group and the Arg^276^ side chain guanidino group. *C,* electron density map of an active site close-up. Representative 2m*F_o_* − D*F_c_* electron density map contoured to 1σ (*blue mesh*) around selected active site residues (*white sticks*), Ni(II) (substituting for Fe(II), *green sphere*), and NOG (*yellow sticks*).

**Figure 7:**
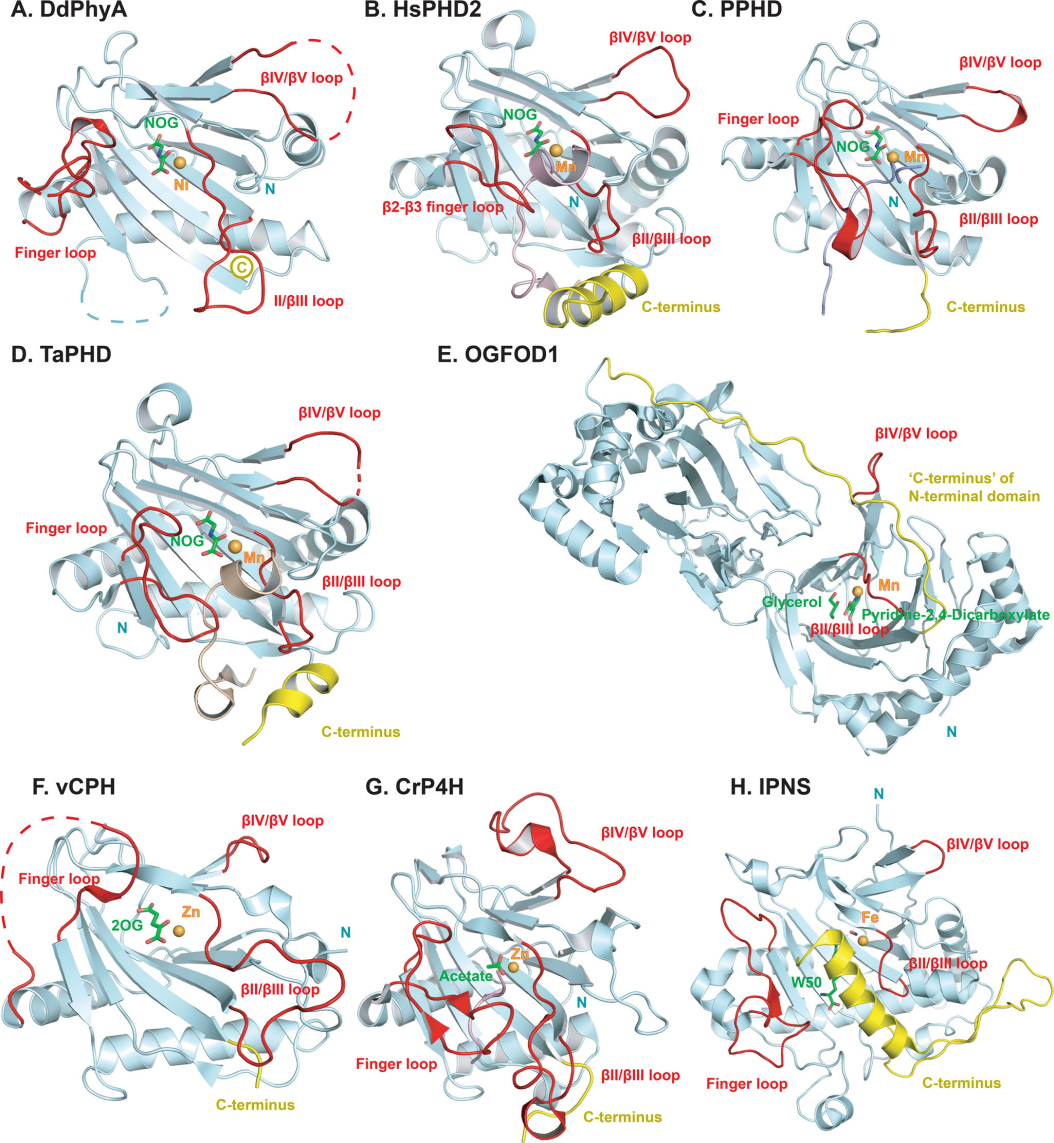
**Comparisons of the finger loop, βII-βIII, and βIV/βV insert loops, and the C-terminal regions in prolyl hydroxylase structures.***A,* DdPhyA (PDB code 6T8M); *B,* HsPHD2 (PDB code 6L7V3HQR); *C,* PPHD (PDB code 4IW3); *D,* TaPHD (PDB code 6F0W); *E,* OGFOD1 (PDB code 4NHY); *F,* vCPH (PDB code 5C5T); *G,* CrP4H (PDB code 3GZE); and *H,* IPNS (PDB code 1W06). The finger loop (which in HsPHD2 links β2 and β3) and βII-βIII, βIV-βV insert loops are in *red*. C-terminal regions following the DSBH are in *yellow*; this region leads to the linker between the two DSBH domains in OGOFD1). Note: the highlighted βIV-βV insert loop has been shown to be involved in substrate binding by some 2OG oxygenase subfamilies, but this is not the case for the prolyl hydroxylase subfamily. Active site metal ion and ligands are shown as a ball and stick.

**Figure 8:**
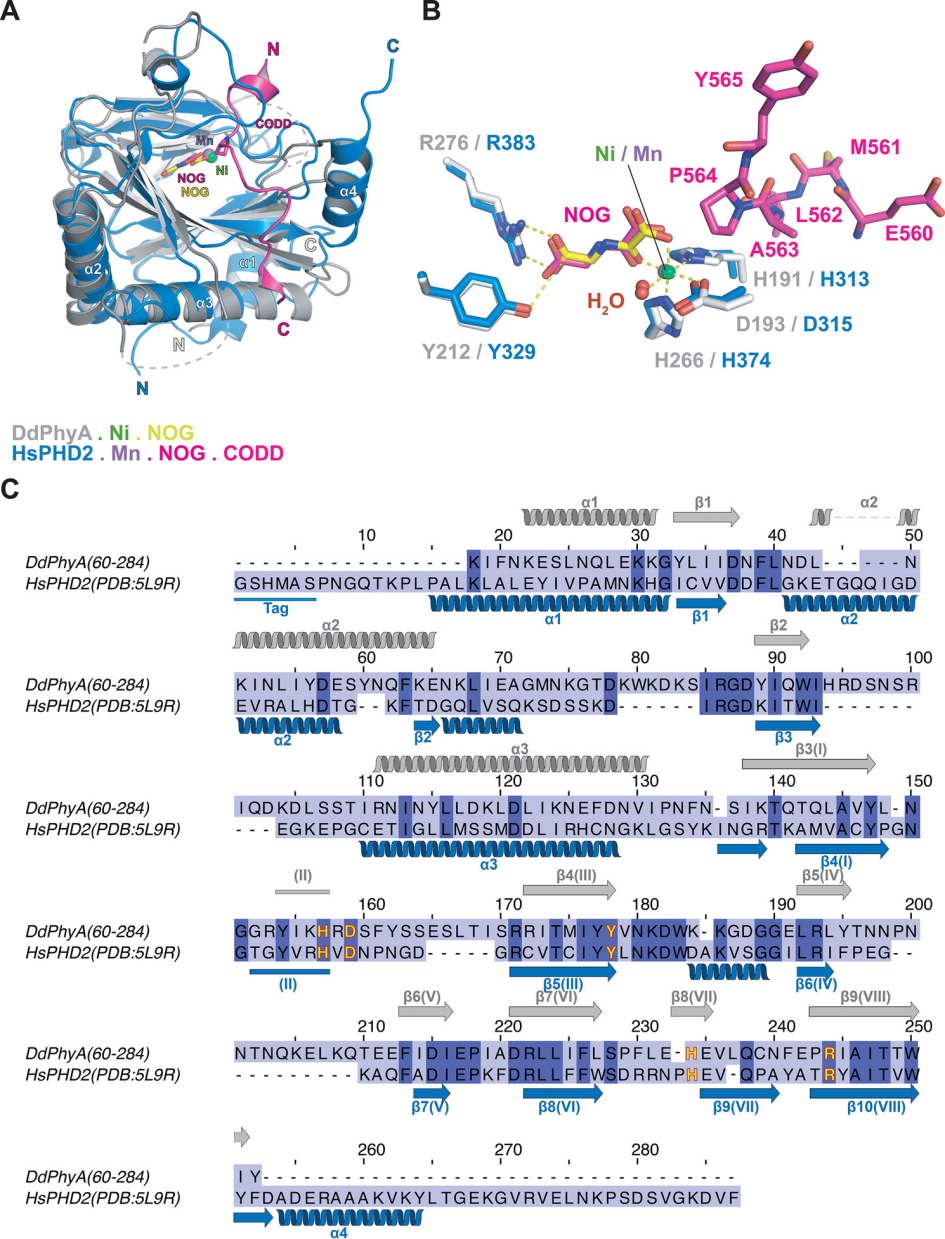
**Comparison of DdPhyA and human PHD2 structures.***A,* superimposition of views from structures of the DdPhyA·Ni·NOG and PHD2·Mn·NOG·HIF-1α CODD (PDB code 3HQR) complexes. *B,* superimposition of active site views. Note that HIF-1α CODD is bound to PHD2, but substrate is not present for DdPhyA. *C,* sequence alignment of DdPhyA(60-284) and the catalytic domain of human PHD2(181-426). Residues involved in Fe(II) binding are in *yellow*.

Consistent with all reported 2OG oxygenase structures, DdPhyA contains an eight stranded “distorted” DSBH (βI-βVIII) core-fold (Fig. 6*A*) (16, 33). Unusually, but not uniquely (*e.g.* the 1-amino-1-cyclopropanecarboxylate oxidase (PDB code 5TCV) (62), the region that is typically β-strand II of the DSBH is random coil. Seven additional β-strands (β3-β9/βI-βVIII) enable formation of a β-sandwich, one end of which forms a deep active site cleft, as occurs with HsPHD2 (16) and most other structurally related 2OG oxygenases (Figs. 7, 8, S9, and S10). Six antiparallel β-strands (β1, β2, β3(I), β4(III), β7(VI), β9(VIII)) form the major β-sheet and four antiparallel β-strands ((II), β5(βIV), β6(V), β8(VII)) form the minor β-sheet of the sandwich. Three N-terminal helices (α1–α3) stabilize the major β-sheet. The structure has three disordered regions (Asn^134^–Leu^142^ (finger loop), Asn^230^–Glu^244^ (β5(IV)/β6(V) loop), and Arg^161^–Arg^174^ ((II)–β4(III) loop)). Each of these disordered loops is likely involved in substrate binding, as is the case for some other 2OG oxygenases (Figure 7, Figure 8, see below) (18). Notably, the C terminus of DdPhyA is much shorter than all other prolyl hydroxylases for which structures are available, *i.e.* it is truncated at the end of β9 (VIII, C-terminal residue: Tyr^284^) (Fig. 7). The absence of a C-terminal helix has mechanistic consequences (see below).

The DdPhyA active site contains three protein metal ion ligands, *i.e.* His^191^, Asp^193^, and His^266^, which form the conserved HXD…H motif present in most, but not all, 2OG oxygenases (Fig. 6*B*) (63). His^191^ is positioned on DSBH II, Asp^193^ on the loop linking II and III of the DSBH, and His^266^ on DSBH VIII (β8). Octahedral coordination of the nickel ion (substituting for iron) is completed by a water molecule and the oxalyl group of NOG, the latter in a bidentate manner. The C-5 carboxylate of NOG is bound via a salt bridge involving both its oxygens with the Arg^276^ guanidino group and via one of its oxygens with the Tyr^212^ alcohol (Fig. 6*B*). The C-5 carboxylate of 2OG likely binds in an analogous manner.

Although there are minor differences, *e.g.* in the precise 2OG methylene/NOG NH positions, the geometry of the metal ion and NOG/2OG-binding elements of DdPhyA are strikingly similar to those observed in HsPHD2 and TaPHD (16) (Figs. 6*B* and 8*B*). The PHD/DdPhyA conservation extends to the metal ion coordination of 2OG/NOG, *i.e.* its C-1 carboxylate is *trans* to the distal histidine (His^266^) of the metal binding HXD…H motif. This conservation is consistent with the low levels of substrate-uncoupled 2OG turnover for DdPhyA and the PHDs, but contrasts with TgPhyA. The presence of electron density for a well-defined water molecule ligating *trans* to the proximal histidine (His^191^) of the metal binding triad is significant. The unusually slow reaction of PHD2 and other animal PHDs including TaPHD, with dioxygen is proposed to enable their hypoxia sensing roles (56, 64, 65, 66, 67). Controlled reaction of the PHDs with dioxygen is proposed to be achieved via tightly regulated translocation of dioxygen from the protein surface to the Fe(II), leading to displacement of a metal ligating water (65). Subsequent generation of a reactive ferryl intermediate adjacent to the substrate C-H bond to be oxidized occurs (48, 50). Superimposition of the DdPhyA·Ni·NOG structure with those of PHD2·Mn·NOG·CODD (Fig. 8, *A* and *B*) and PPHD·Mn·NOG·EF-Tu (Figs. S10, A and B, and S11) implies the DdPhyA active site region can accommodate a Skp1 substrate in a similar mode to its biochemical orthologues, including with respect to the proline-residue C-4 *endo* conformation observed in the enzyme-substrate complexes of HsPHD2 (50) and PPHD (33), which as with DdPhyA leads to a *trans*-4-hydroxyprolyl product (Figs. 1, 8*B*, and S11*B*).

The overall conservation of active site geometry in DdPhyA and HsPHD2/TaPHD, all of which have assigned dioxygen sensing roles (9, 33, 48), is striking. The detailed factors regulating the rate of dioxygen translocation to prolyl hydroxylase active sites, and rate of reaction once there, are not fully understood and may be affected by subtle factors, *e.g.* protonation states, so enabling differences in kinetics (65, 68).

The available evidence is that 2OG oxygenases typically use one or more flexible loop(s) to bind macromolecular substrates, likely by an induced-fit process (Fig. 7) (18, 19, 20, 21). By contrast 2OG oxygenases and structurally related enzymes that accept small molecule substrates, tend to have shorter loops, *e.g.* an extended finger loop linking strands β2–β3 in HsPHD2 is much shorter in IPNS (Fig. 7*H*). By comparing the DdPhyA structure with those of prolyl hydroxylases in complex with substrates, including HsPHD2 and TaPHD, it can be inferred that the partially disordered finger loop (residues 102-124 in DdPhyA) and the βII–βIII (residues 191-205) region are similarly involved in substrate binding by DdPhyA (Fig. 7). Thus, in this regard, substrate binding by DdPhyA and likely TgPhyA is similar to the PHDs and some, but not all, other studied prolyl hydroxylases (Fig. 7).

By contrast, the C terminus of DdPhyA is strikingly unusual, including compared with the PHDs, in that it terminates immediately after β9(VIII). The vast majority of related 2OG oxygenases for which structures are available have structural features extending beyond βVIII (18, 19, 20, 21), these can range from only a few residues to one or more α-helices or even entire domains (Fig. 7). A C-terminal α-helix is present in the PHDs and many 2OG oxygenases/related enzymes, not acting on proteins, *e.g.* IPNS (Fig. 7*H*) (18, 21). However, a C-terminal helix is not present in other studied types of prolyl hydroxylases (19) (Fig. 7, *C* and *E–G*), including procollagen-type prolyl hydroxylases (69, 70), OGFOD1 (68), and PPHD (33). In some cases, including the JmjC subfamily 2OG-dependent oxygenases, such as FIH (71), the C terminus is extended in a manner enabling dimerization. The dimer interface can be involved in substrate binding as with FIH, where it may have a role in unfolding ankyrin repeat domain substrates (the HIF-α, CTAD substrate of FIH (55, 72), like the ODDs, is also intrinsically disordered) (49, 71, 73). The apparent conservation of a C-terminal α-helix in the animal PHDs (9) may be important when considering evidence for reported non HIF-α substrates of the PHDs, some of which are apparently buried within folded regions.

Previous work, principally with HsPHD2 (48, 74), but also with other 2OG oxygenases, including deacetoxycephalosporin C synthase (DAOCS) (75), has shown that the C terminus can be involved in substrate binding and productive coupling of 2OG turnover to substrate oxidation (64, 74). Notably, TgPhyA (and TgPhyB) has a longer C terminus than DdPhyA, even though it accepts the same Skp1 substrate. It may be that the difference in the C-terminal regions between DdPhyA and TgPhyA is in part responsible for their differences in kinetic properties, including the observation that TgPhyA catalyzes substantially more substrate uncoupled turnover of 2OG than DdPhyA. Consistent with this proposal, previous studies, including on HsPHD2, have shown that shortening the C terminus can affect substrate binding and promote uncoupled 2OG turnover (74). The results for DdPhyA, however, demonstrate that an extended prolyl hydroxylase C terminus is not necessary for efficient coupling of 2OG turnover with substrate oxidation.

## Discussion

Despite their different types of substrate, *i.e.* the HIF-α ODDs and Skp1, the conserved role of PHD-type prolyl hydroxylases in hypoxia sensing in both animals and simpler organisms is striking from an evolutionary perspective. Unlike HIF-α ODD substrates of the HsPHDs and HIF-α CTAD substrates of FIH, but like the bacterial prolyl hydroxylase, PPHD, which acts on EF-Tu and ankyrin repeat domain FIH substrates, the Skp1 substrates of DdPhyA and TgPhyA have ordered core-folds. Most previous kinetic analyses on eukaryotic prolyl hydroxylases acting on proteins, including the HsPHDs, have employed peptide fragments of the intact protein substrates (74). The work presented here employed full-length DdPhyA and TgPhyA Skp1 protein substrates and directly monitored the extent of hydroxylation by MS.

The results reveal substantial differences in the *in vitro* kinetic properties of DdPhyA and TgPhyA, both with respect to each other and in comparison, with human PHD2 (Figure 3, Figure 4, Figure 5 and Table 1, Table 2). Notably, the $K_{m}^{\text{app}}$ values for the Skp1 substrates of both DdPhyA and TgPhyA are relatively high (*i.e.* ∼350 μm for DdPhyA and ∼200 μm for TgPhyA) compared with the values reported for the HsPHDs/TaPHD and HIF-α fragment (ODD) substrates using comparable methods (*i.e.* ∼10-50 μm HsPHD2 with HIF-1α/2α NODD/CODD) (54), although comparable with those reported for FIH catalyzed HIF-α CTAD hydroxylation (54). A caveat of our work is that the Skp1 proteins were produced in *Escherichia coli* and as a result any endogenous PTMs that may affect activity would be absent. However, there are no reports on DdSkp1/TgSkp1 PTMs other than prolyl hydroxylation and hydroxyproline glycosylation.

The DdPhyA $K_{m}^{\text{app}}$ for 2OG was in the same range as reported for other prolyl hydroxylases (∼35 μm). However, it was not possible to measure an accurate TgPhyA $K_{m}^{\text{app}}$ value for 2OG due to a lack of saturation. Although in the presence of a 5-fold excess of 2OG, Skp1 substrate undergoes efficient hydroxylation (as observed by MS). It is possible that the reason for the lack of saturation is connected to the high level of 2OG turnover catalyzed by TgPhyA in the absence of Skp1 substrate. By contrast, DdPhyA, like HsPHD2 (64), only catalyzed low levels of 2OG turnover in the absence of substrate. In this regard structural studies on TgPhyA, which manifests different kinetic properties to DdPhyA and HsPHD2 (74, 82), are of interest.

The biological significance of the high level of uncoupled 2OG turnover by TgPhyA, if any, is unclear. Whether or not catalysis by sets of 2OG oxygenases directly affects 2OG/succinate concentrations in a physiologically relevant manner in cells is presently an open question. Mutations to genes encoding for TCA cycle and related enzymes can lead to substantial changes in concentrations of metabolites, such as 2OG, succinate, and 2-hydroxyglutarate (76, 77). In turn this may lead to impaired catalysis by 2OG oxygenases involved in, for example, the hypoxic response and the modification of the epigenetic machinery (*e.g.* KDMs), with potential promotion of tumorigenesis (78, 79). It is also possible that 2OG (or dioxygen) consumption by some 2OG oxygenases limits its local availability for use by other 2OG utilizing enzymes, including other 2OG oxygenases. Differences in the kinetic properties of some 2OG oxygenases may make them especially susceptible to changes in 2OG availability, *e.g.* γ-butyrobetaine hydroxylase (BBOX1) has an unusually high $K_{m}^{\text{app}}$ for 2OG, a property that may be linked to its role in carnitine biosynthesis and lipid metabolism (80). Hence, the observed high uncoupled 2OG turnover for TgPhyA in the absence of Skp1 supports further exploration of potential interactions between the prolyl-hydroxylases of *T. gondii*, TgPhyA and TgPhyB, and TCA cycle and related metabolism involving 2OG, dioxygen, and succinate.

The high $K_{m}^{\text{app}}$ value of dioxygen for DdPhyA is close to the value reported for HsPHD2 (56, 81), whereas TgPhyA was much more active at lower dioxygen concentrations. Our results are consistent with those reported, notably that TgPhyA has a high affinity for dioxygen (82). The high $K_{m}^{\text{app}}$ value for dioxygen for DdPhyA is consistent with its proposed hypoxia sensing role in *D. discoideum* (58). Interestingly, *T. gondii* TgPhyA has a paralogue, TgPhyB, the substrate(s) of which is not yet identified; however, TgPhyB is proposed to be a sensor for high dioxygen levels. Like PPHD in *Pseudomonas putida*, TgPhyB is linked to translation regulation in a manner involving adhesin abundance and the elongation factor, eEF2 (32, 47). Once biochemical assays for TgPhyB have been established, it will be of interest to compare its kinetic properties with TgPhyA and other PHD orthologues.

Crystallographic analyses on DdPhyA reveal strong conservation of the active site structure in the region of the Fe(II)-binding site with PHD homologs, including with respect to the coordination chemistry (Fig. 6, *B* and *C*), *i.e.* the binding mode of 2OG (based on the 2OG analog, NOG) and an apparently tightly bound water molecule, with PHD2 and PPHD (Figs. 8, S10, and S11) (16, 33). Despite these and other active site similarities, we observed no cross-reactivity in terms of the (WT) substrates accepted by the different types of eukaryotic PHDs (Table 1, Table 2). Interestingly, however, the human Skp1(E147P) variant is a substrate for both DdPhyA and TgPhyA (Fig. 4 and Table 1) and human Skp1 inhibits the substantial 2OG turnover catalyzed by TgPhyA in the absence of substrate. These observations imply not only conservation of the overall Skp1-fold in eukaryotes, but perhaps more surprisingly, of its dynamic interactions with both DdPhyA and TgPhyA.

Although a structure of DdPhyA in complex with its Skp1 substrate is not available, comparison of the DdPhyA structure with those of other prolyl hydroxylases and 2OG oxygenases (Figs. S10 and S11), including PHD2 and PPHD in complex with their substrates (33, 50), reveals that the finger loop and the βIV-βV insert loop are very likely involved in induced-fit type substrate binding (Figs. 8, S10, and S11). The substantial inhibitory activity of mutant DdSkp1(P143A), is consistent with determinants for Skp1 recognition lying outside of the active site region (33, 52, 53). Free DdSkp1 has a well-ordered fold over its first 122 residues that is near identical to its structure in complex with F-box proteins (84), but evidence indicates that the C-terminal 40 residues, which include the hydroxylated Pro^143^ in its central region and corresponds to the longest peptide tested, is disordered and subject to reorganization by glycosylation (38, 85). Thus, the dynamics of substrate binding by DdPhyA and TgPhyA are likely different not only with respect to the PHDs but also with respect to bacterial orthologues, such as PPHD. The ODD PHD substrates are substantially disordered (49), whereas EF-Tu and Skp1 have well-ordered folds, although the surface loops on which the target prolyl residues exist are dynamic (33). Although further work is required, it may be that, along with tight binding of Fe(II) and 2OG (49), the unstructured nature of HIF−α substrate is an evolved property that helps enable the PHDs to focus on hypoxia sensing or which may enable fine tuning of hypoxia sensing in animals. In this regard, animal FIH is notable because it accepts both ordered ankyrin repeat domain substrates, as well as disordered HIF-α CTAD, substrates.

A striking feature of DdPhyA is that its C terminus is curtailed at the end of βVIII in the core DSBH-fold (Fig. 6*A*). This contrasts not only with the PHDs and PPHD, but with TgPhyA (and TgPhyB), whose sequence indicates that their C termini are more typical, being extended by 10-11 residues compared with DdPhyA (Fig. S1). In the case of the PHDs and structurally related 2OG oxygenases the C-terminal tail has been shown to have roles both in substrate binding/selectivity and in coupling of 2OG and substrate oxidation (64, 75, 86). The shorter C terminus of DdPhyA and evidence that C terminally appended epitope tag peptide enables complementation of catalysis and O_2_ sensing (57) reveals that a C-terminal tail is not essential for the aforementioned processes. This knowledge may be useful, including in ongoing studies concerning engineering of 2OG oxygenases for biocatalytic purposes.

The overall results provide structural and kinetics insights into how a conserved hypoxia-sensing mechanism employing prolyl hydroxylation has evolved, likely starting from microbial origins. The HIF system is presently being explored for therapeutic benefit via inhibition of the HsPHDs (12). Knowledge on the evolution of the HsPHDs may help inform on poorly understood aspects of PHD/HIF biology in humans and thus help guide therapeutic strategies, with one important aspect being the validation or otherwise of nonHIF PHD substrates. The assays reported here will be useful for the identification of inhibitors of TgPhyA and DdPhyA (13). Given that *T. gondii* is a widespread human parasite (87), inhibitors of its 2OG oxygenases, including TgPhyA, may be of medicinal value as well as of use as functional probes for use in research. The structural studies reported here suggest that with optimization that the scaffolds present in current HsPHD inhibitors may be suited for TgPhyA inhibition.

## Experimental procedures

### Purification of DdPhyA and DdPhyA(60-284)

DNA encoding for N terminally His_6_-ZBasic-tagged *D. discoideum* full-length DdPhyA (aa 1-284, DdPhyA) was cloned into the pNIC-ZBasic vector (88). Recombinant DdPhyA protein was produced in *E. coli* BL21 GOLD (DE3) cells. The cells were grown at 37 °C in 2× tryptone/yeast extract (2TY) media supplemented with 10 μg/ml of kanamycin to an *A*_600_ of 1.2. Expression was initiated by 0.2 mm isopropyl β-d-thiogalactopyranoside. Growth was continued at 18 °C for 16–18 h; cells were harvested by centrifugation (10,000 × *g*, 8 min) and stored at −80 °C. The cell pellets were resuspended in lysis buffer (50 mm HEPES, pH 7.6, 500 mm NaCl, 5 mm imidazole, and DNase I (bovine pancreas, grade II, Roche)). Cells were lysed by sonication on ice (Sonics Vibracell VCX750 Ultrasonic Cell Disruptor), 60% amplitude, 10 cycles of 30 s sonication with 40 s rest between each cycle. The cell lysates were loaded onto a 5-ml HisTrap column (GE Healthcare), equilibrated with binding buffer (50 mm HEPES, pH 7.6, 500 mm NaCl, 5 mm imidazole) and separated using an AKTA purifier (GE Healthcare). The column was washed with wash buffer (50 mm HEPES, pH 7.6, 500 mm NaCl, 30 mm imidazole). His_6_-ZBasic-DdPhyA was eluted with elution buffer (50 mm HEPES, pH 7.6, 500 mm NaCl, 500 mm imidazole) using a linear gradient (0–100%). Based on the UV-trace (280 nm) and SDS-PAGE, fractions containing purified His_6_-ZBasic-DdPhyA were pooled. The combined fractions were then treated with the tobacco etch virus protease (1/50 mg of target protein) and 50 mm EDTA overnight at 4 °C to cleave the His_6_-ZBasic tag and remove metal ions. The solution was concentrated to 2.5 ml using a membrane filter (30-kDa molecular mass cutoff). The concentrated protein was exchanged into binding buffer (50 mm HEPES, pH 7.6, 500 mm NaCl, 5 mm imidazole) using a PD-10 desalting column (GE Healthcare). To remove the tobacco etch virus protease, the solution was then loaded onto a 5-ml HisTrap column (GE Healthcare). The DdPhyA containing fractions were concentrated using a membrane filter (30-kDa molecular mass cutoff membrane), then loaded onto a Superdex 75 size-exclusion column (GE Healthcare), pre-equilibrated with size-exclusion buffer (50 mm HEPES, pH 7.6). The desired fractions (based on UV-trace and SDS-PAGE analyses) were concentrated to 25-30 mg/ml, then frozen in liquid nitrogen and stored at −80 °C. Truncated DdPhyA (DdPhyA(60-286)) used for crystallography was purified by the same purification procedure used for WT protein, except with use of a different storage buffer (50 mm HEPES, pH 7.6, 50 mm NaCl). Procedures for purifying other enzymes/proteins are described under the Supporting information.

### Hydroxylation and time course assays

Hydroxylation assays were performed in triplicate under normoxic conditions using liquid chromatography electrospray MS (LC-ESI-MS) (Figure 3, Figure 4). Conditions: 1 μm TgPhyA or DdPhyA, 100 μm full-length DdSkp1 or full-length TgSkp1 substrate, 50 μm (NH_4_)_2_Fe(II)(SO_4_)_2_ (Sigma-Aldrich), 1 mm sodium l-ascorbate (Sigma-Aldrich), and 500 μm 2-oxoglutarate disodium salt (Sigma-Aldrich) in HEPES (100 mm), pH 7.6. Mixtures were incubated at 37 °C for 1 h, or at specific time points for time course assays, and quenched using an equal volume of 1% (v/v) aqueous formic acid (Sigma-Aldrich). Reaction mixtures were analyzed using a Xevo G2-S Q-TOF mass spectrometer equipped with an electrospray source (Waters®) coupled to a Waters® ACQUITY UPLC System, unless otherwise specified. Instrument control and data processing were performed using MassLynx V4.1 software. An Aeris^TM^ 3.6-μm WIDEPORE C4 200 Å (Phenomenex) 4.6 × 50 mm column was used for separation.

### Kinetic assays

Kinetic assays were performed under normoxic/atmospheric conditions using LC-ESI-MS as described above, except for the *K_m_* determination of 2OG dependence of DdPhyA-mediated DdSkp1 hydroxylation and the O_2_ dependence of TgPhyA-mediated TgSkp1 hydroxylation, which were analyzed by solid-phase extraction coupled to MS (RapidFire MS, see below). *K_m_* values were determined by fitting to the Michaelis-Menten equation in GraphPad Prism 5.

Initial time course assays were conducted and analyzed prior to kinetic studies to test whether the reactions were in the linear range during kinetic studies. Note that in the case of TgPhyA, more than one +16 Da increment of TgSkp1 was observed on prolonged incubation and in no-enzyme controls, a +16 Da TgSkp1 peak was observed. However, nonenzymatic TgSkp1 hydroxylation was below the detection limits within the first 30 min of reaction. Because the time course of TgPhyA catalyzed hydroxylation was linear within the first 2 min, apparent $K_{m}^{\text{app}}$ measurements with TgSkp1 were quenched after 2 min. Assays were carried out in triplicate. To investigate the O_2_ dependence of the reaction, a mass-flow controller (Brooks Instrument) was used to vary the O_2_ concentration (56). Thus, 94 μl of full-length DdSkp1 or full-length TgSkp1 substrate solutions were equilibrated for 10 min at 37 °C with O_2_/N_2_ in the desired ratio in rubber septa-sealed glass vials (Sigma-Aldrich), before addition of 2OG, l-ascorbate, Fe(II), and enzyme using gas-tight Hamilton syringes, to give the desired final concentrations. Conditions for the individual kinetic analyses are given under the Supporting information.

### Solid phase extraction MS analyses

The 2OG dependence of DdPhyA-mediated DdSkp1 hydroxylation and O_2_ dependence of TgPhyA-mediated TgSkp1 hydroxylation were assayed in duplicate under normoxic conditions using a RapidFire RF360 high throughput sampling robot (Agilent) connected to an Agilent 6530 QTOF spectrometer (89). When varying 2OG, reaction mixtures (1 μm DdPhyA, 100 μm full-length DdSkp1, 50 μm Fe(II), 1 mm l-ascorbic acid, and 2OG (20, 50, 100, 200, and 300 μm)) were incubated for 6 min at 37 °C, then quenched with an equal volume of 1% (v/v) aqueous formic acid. When varying O_2_%, mixtures (1 μm TgPhyA, 600 μm full-length TgSkp1, 500 μm 2OG, 50 μm Fe(II), 1 mm l-ascorbic acid) equilibrated at different dioxygen concentrations (0–30% oxygen in air) were incubated for 2 min at 37 °C, then quenched with an equal volume of 1% (v/v) formic acid. Samples were diluted 1:100 with 50 mm Tris-HCl, pH 7.5, 50 mm NaCl and then aspirated under vacuum (500 ms) and loaded onto a C4 SPE cartridge. The cartridge was washed for 5000 ms with 0.1% (v/v) aqueous formic acid at a flow rate of 1.5 ml/min; the protein was eluted with 85% (*v/v*) aqueous acetonitrile and 0.1% (v/v) formic acid for 5000 ms; flow rate: 1.25 ml/min. The SPE cartridge was then re-equilibrated with 0.1% (v/v) aqueous formic acid for 800 ms; flow rate: 1.5 ml/min. Skp1 substrate hydroxylation was measured in the positive ion ESI mode (drying gas temperature: 280 °C, drying gas flow rate: 13 liters/min, nebulizer gas pressure: 40 p.s.i., cone voltage: 200 V). Extracted ion chromatogram data were integrated and the % conversion was calculated using: 100 × hydroxylated/(hydroxylated + nonhydroxylated). *K_m_* was determined by fitting to the Michaelis-Menten equation in GraphPad Prism 5.

### Trypsin digestion and MS/MS studies

In-solution digestion was carried out by reaction with trypsin (20 ng/μl) in 50 mm NH_4_HCO_3_ (Sigma-Aldrich) for 30 min (90, 91). Prior to digestion, samples were reduced (45 min, 10 mm DTT, 100 mm NH_4_HCO_3_) and alkylated (30 min of 55 mm iodoacetamide in 100 mm NH_4_HCO_3_). In-gel digestion was carried out under the same procedure with the excised band after SDS-PAGE gel separation (92). Peptides were analyzed using a NanoACQUITY-UPLC system (Waters®) machine coupled to an Orbitrap Elite™ mass spectrometer (Thermo Fisher Scientific™) (93) possessing an EASY-Spray nano-electrospray ion source (Thermo Fisher Scientific™). Peptides were injected onto a guard column (75 μm internal diameter × 20 mm, Acclaim Pepmap100 C18, 3 μm, 120 Å) using solvent A (Milli-Q water, 0.1% (v/v) aqueous formic acid). They were separated using an EASY-spray Acclaim PepMap® analytical column (75 μm inner diameter × 15 mm, RSLC C18, 3 μm, 100 Å) using a linear gradient (68 min, 3 to 97% (v/v) solvent B (20% Milli-Q water, 80% acetonitrile, 0.1% (v/v) formic acid), flow rate: 300 nl/min). Data-dependent MS acquisition full-scan MS spectra were collected (scan range 350-1500 *m/z*, resolution 120,000, AGC target 1E6, maximum injection time 100 ms). The 10 most intense peaks were selected for collision-induced dissociation fragmentation at 35% of the normalized collision energy. Collision-induced dissociation spectra were acquired in the Iontrap (AGC target 1e3, maximum injection time 100 ms). Results were analyzed using PEAKS® 8.0 (Bioinformatics Solutions Inc., Waterloo, Canada) (94).

### 2OG turnover assays by ^1^H NMR

^1^H NMR spectroscopy was used to monitor the conversion of 2OG to succinate during hydroxylation, as catalyzed by DdPhyA, TgPhyA, and PHD2. For details regarding the substrates used, see Supporting information. Proteins were buffer exchanged into 50 mm Tris-D_11_, pH 7.5, prior to assays. Unless otherwise stated, assay mixtures contained 20 μm enzyme, 50 μm Fe(II), 500 μm 2OG, 400 μm Skp1 substrate, and 1 mm ascorbate buffered with 50 mm Tris-_D11_, pH 7.5, 10% H_2_O, 90% (v/v) D_2_O. Reactions were initiated by addition of 2OG at room temperature under normoxic conditions. NMR spectra were recorded using a Bruker AVIII 700 MHz NMR spectrometer equipped with a 5-mm inverse cryoprobe using 3-mm MATCH NMR tubes (Cortecnet). ^1^H excitation sculpting suppression NMR spectra were obtained using 64 scans. Changes in integration of the peaks of interest (the 2OG methylene triplet at 2.22 ppm and succinate methylene singlet at 2.18 ppm) were monitored and plotted, as reported (60, 61). Data were processed using Bruker 3.1 software.

### 2OG binding experiments by ^1^H-edited CPMG

Typical experimental parameters for CPMG NMR spectroscopy were as follows: total echo time, 40 ms; relaxation delay, 2 s; and number of transients, 264. The PROJECT-CPMG sequence (90°x−(τ − 180°y − τ − 90°y − τ − 180°y − τ)*_n_*−acq) was applied. Water suppression was achieved by pre-saturation. Assay mixtures contained 50 μm 2OG, in the presence or absence of 50 μm Skp1 (as indicated in the Supporting information) buffered with 50 mm Tris-D_11_, pH 7.5, 10% H_2_O, 90% (v/v) D_2_O. Data were recorded with a Bruker AVIII 700 MHz NMR spectrometer equipped with a 5-mm inverse cryoprobe using 5-mm NMR tubes (60, 61).

### NMR assignment of hydroxylation regiochemistry

Assay mixtures contained DdPhyA (10 μm) or TgPhyA (10 μm), DdSkp1 peptide (100 μm) or TgSkp1 peptide (100 μm), (NH_4_)_2_Fe(II)(SO_4_)_2_ (10 μm), sodium l-ascorbate (100 μm), and 2OG (50 μm), in HEPES (100 mm), pH 7.6. Reactions were incubated at 37 °C for 1 h under normoxic conditions and then quenched using an equal volume of 1% (v/v) formic acid. The hydroxylated peptides were partially purified using a Sep-Pak Cartridge (Waters®). Samples were dried under vacuum, then dissolved in D_2_O to a concentration of 4 mm prior to 2-D total correlation spectroscopy ^1^H analysis using a Bruker AV700 MHz NMR spectrometer equipped with an inverse TCI ^1^H/^13^C/^15^N CryoProbe. 4 scans were acquired at the resolution of 512 data points in the indirect detected dimension. Mixing time was 100 ms.

### Differential scanning fluorimetry (T_m_ shift) assays

*T_m_* shift assays were used to investigate the optimal conditions (Table S3) of buffer, pH, metal, and cofactors that stabilize the protein. Different metals (Mn^2+^, Fe^2+^, Zn^2+^, Mg^2+^, Ni^2+^, Co^2+^, and Cd^2+^) and cofactors 2OG or NOG were screened in the *T_m_* shift assays for both DdPhyA and TgPhyA using a CFX96 Touch™ Real-Time PCR Detection System (Bio-Rad). The purified enzymes were mixed with the appropriate metal chloride salt and cofactors. Nonmetal or noncofactor conditions were used as controls. The SYPRO Orange dye (Sigma-Aldrich) was added last. The mixed solutions were incubated at room temperature for 10 min before measurements were taken. Fluorescence readings (in triplicate) were taken every 1 °C increment from 25 to 80 °C. The data were fitted to Boltzmann curves using GraphPad Prism 5.

### Crystallization and structure determination of DdPhyA(60-284)

Crystallization trials employed commercially available broad screens followed by optimization. DdPhyA (residues 60-284) of high purity (≥95%, based on SDS-PAGE analysis) at a concentration of 23 mg/ml was used. DdPhyA(60-284)·Ni(II)·NOG co-crystals (23 mg/ml of DdPhyA(60-284), 2 mm NiCl_2_, 2 mm NOG) were formed by first incubating protein stock with Ni(II) and NOG on ice for ∼1 h. A Phoenix™ RE crystallization liquid handler (Art Robbins Instruments, USA) was used in crystallization trials. Crystals were grown by the sitting drop vapor diffusion method in 96-well low-profile Intelliplates (Art Robbins Instruments, USA) (drop size 200-300 nl) at room temperature. Crystals were obtained from a commercial broad screen (Hampton Research Crystal Screen HT, number 35). Proteins were in 50 mm HEPES, 100 mm NaCl, pH 7.6, at 25 °C. The optimized crystallization condition was: 0.1 m HEPES, pH 7.5, 17.5–23% (*w/v*) PEG 6000, 0.2 m NaCl, 2 mm NiCl_2_, 6% (v/v) glycerol. Crystals were cryo-protected using reservoir solution diluted to 25% (*v/v*) glycerol and cryo-cooled by plunging into liquid nitrogen. Diffraction data were collected at 100 K at the Diamond Light Source beamline I03 (λ 0.97623 Å) and processed using XDS in space group *C*222_1_ to 2.0 Å resolution (95, 96) (Table 3). The DdPhyA(60-284) structure was solved by molecular replacement using PHASER, which located 3 molecules in the asymmetric unit using an ensemble search model based on the PPHD (PDB code 4J25) and PHD2 (PDB code 5L9R) structures. Iterative cycles model building and refinement using COOT and CNS or PHENIX continued until *R*_factor_ and *R*_free_ no longer converged (97, 98, 99, 100). MOLPROBITY was used to monitor the geometric quality of the models between refinement cycles (101). Water molecules were added to 2mF_o_-DF_c_ electron density map peaks (>1.5 σ) that were within hydrogen bonding distance to protein with reasonable geometry.

## Data availability

Coordinates and structure factors for the DdPhyA structure are deposited in the RCSB Protein Data Bank as ID 6T8M. All other data are within the manuscript.
