# Supplementary material for: Biochemical and biophysical analyses of hypoxia sensing prolyl hydroxylases from Dictyostelium discoideum and Toxoplasma gondii
Source: J Biol Chem. 2021 Jan 13;295(49):16545–61. doi: 10.1074/jbc.RA120.013998 (PMC7864055; doi:10.1074/jbc.RA120.013998)

Supporting Information

Biochemical and biophysical analyses of hypoxia sensing prolyl hydroxylases from *Dictyostelium discoideum* and *Toxoplasma gondii*

Tongri Liu[1], Martine I. Abboud[1], Rasheduzzaman Chowdhury[1], Anthony Tumber[1], Adam P. Hardy[1], Kerstin Lippl, Christopher T. Lohans[1], Elisabete Pires[1], James Wickens[1], Michael A. McDonough[1], Christopher West[2], Christopher J. Schofield[1]

Table of Contents

PROTEIN PURIFICATION METHODS 2

EXPRESSION AND PURIFICATION OF TGPHYA 2

EXPRESSION AND PURIFICATION OF HIS_6-DDSKP1 2

EXPRESSION AND PURIFICATION OF HIS_6-TGSKP1 3

EXPRESSION AND PURIFICATION OF HSSKP1-HIS_6 AND HSSKP1-HIS_6 E147P 4

EXPRESSION AND PURIFICATION OF HSPHD2 4

KINETIC ASSAY CONDITIONS 6

DDPHYA KINETIC ASSAYS 6

Assay conditions for DdPhyA varying full-length DdSkp1 substrate concentration: 6

Assay conditions for DdPhyA varying 2OG concentration: 6

Assay conditions for DdPhyA varying O_2 concentration: 6

TGPHYA KINETICS 6

Assay conditions for TgPhyA varying the full-length TgSkp1 substrate concentration: 6

Assay conditions for TgPhyA varying 2OG concentration: 6

Assay conditions for TgPhyA varying the O_2 concentration: 6

Table S1. Peptides used in this study. 7

FIGURES AND TABLES 8

FIGURE S1. DDPHYA, PPHD (PDB 4J25), TGPHYA (GENE ID: TGGT1_232960), AND TGPHYB (GENBANK: KYF42913.1) SEQUENCES. 8

FIGURE S2. SEQUENCE ALIGNMENT OF PEPTIDE SEQUENCES OBTAINED BY NANO LC-MS/MS WITH DDSKP1 AND TGSKP1 SEQUENCE. 9

FIGURE S3. ASSIGNMENT OF MODIFICATION SITES OF DDSKP1 BY DDPHYA AS DETERMINED BY LC COUPLED TO TANDEM MS (NANO MS/MS). 10

FIGURE S4. ASSIGNMENT OF MODIFICATION SITES OF TGSKP1 BY TGPHYA AS DETERMINED BY LC COUPLED TO TANDEM MS (NANO MS/MS). 11

FIGURE S5. NMR ANALYSIS OF THE PROLYL-HYDROXYLATION REGIOCHEMISTRY OF DDPHYA AND TGPHYA. 12

FIGURE S6. ANALYSES OF 2OG TURNOVER TO SUCCINATE BY ^{1}H NMR. 13

FIGURE S7. ^{1}H NMR ANALYSES OF 2OG TURNOVER TO SUCCINATE. 14

TABLE S2. 2OG TURNOVER ASSAY CONDITIONS. 14

FIGURE S8. ^{1}H-EDITED CPMG STUDIES ON THE PROPOSED BINDING OF 2OG TO SKP1. 15

FIGURE S9. T_M SHIFT ASSAYS WITH (A) DDPHYA OR (B) TGPHYA. 16

TABLE S3. ASSAY CONDITIONS FOR THE T_M SHIFT ASSAYS WITH DDPHYA AND TGPHYA. 16

FIGURE S10. COMPARISON OF DDPHYA STRUCTURE WITH THOSE OF OTHER PROLYL HYDROXYLASES (COMPLEXED WITH SUBSTRATES). 17

FIGURE S11. COMPARISON OF DDPHYA AND PSEUDOMONAS PUTIDA PPHD STRUCTURES. 18

Protein Purification Methods

Expression and Purification of TgPhyA

DNA encoding for *N*-terminally His_6-ZBasic-tagged *Toxoplasma gondii* full-length TgPhyA (aa 1-271, TgPhyA) was cloned into the pNIC-ZBasic vector (88). Recombinant TgPhyA protein was produced in *Escherichia coli* BL21 GOLD (DE3) cells. The cells were grown at 37 °C in 2x tryptone/yeast extract (2TY) media supplemented with 10 μg/ml kanamycin to an OD_{600} = 1.2. Expression was initiated by addition of 0.2 mM isopropyl β-D-1-thiogalactopyranoside (IPTG). Growth was continued at 18 °C for another 16-18 hours; cells were harvested by centrifugation (10,000g, 8 minutes) and stored at -80 °C. The cell pellets were resuspended in lysis buffer (50 mM HEPES pH 7.6, 500 mM NaCl, 5 mM imidazole, and DNaseI (bovine pancreas, grade II, Roche)). Cells were lysed by sonication on ice (Sonics Vibracell VCX750 Ultrasonic Cell Disruptor) 60 % amplitude, 10 cycles of 30 s sonication with 40 s rest between each cycle. The lysates were filtered using a Minisart 0.45 μm syringe filter (Sartorius Stedim Biotech) then loaded onto a 5 ml HisTrap column (GE Healthcare), that had been equilibrated with binding buffer (50 mM HEPES pH 7.6, 500 mM NaCl, 5 mM imidazole) and separated using an AKTA purifier (GE Healthcare) carried out at 4 °C with a flow rate of 1 ml/min. The column was washed with wash buffer (50 mM HEPES pH 7.6, 500 mM NaCl, 30 mM imidazole). His_6-ZBasic-TgPhyA was eluted with elution buffer (50 mM HEPES pH 7.6, 500 mM NaCl, 500 mM imidazole) using a linear gradient (0-100 %). Based on the UV-trace (280 nm) and sodium dodecyl sulfate-polyacrylamide gel electrophoresis (SDS-PAGE), fractions containing purified His_6-ZBasic-TgPhyA were pooled. The combined fractions were then treated with the TEV protease (1 mg / 50 mg target protein) and 50 mM ethylenediaminetetraacetic acid (EDTA) overnight at 4 °C to cleave the His_6-ZBasic tag and remove metal ions. The protein solution was then concentrated to 2.5 ml using a 30 kDa MW cut-off 15 ml concentrator (Amicon®). The concentrated protein was exchanged into binding buffer (50 mM HEPES pH 7.6, 500 mM NaCl, 5 mM imidazole) using a PD-10 desalting column (GE Healthcare). To remove the TEV protease, the solution was then loaded onto a 5 ml HisTrap column (GE Healthcare). The flow-through containing TgPhyA was collected and concentrated using 30 kDa MW cut-off 15 ml concentrator (Amicon®), then loaded onto a Superdex 75 size-exclusion column (GE Healthcare), which had been pre-equilibrated with size-exclusion buffer (50 mM HEPES pH 7.6), for further purification. The desired fractions (based on UV-trace and SDS-PAGE analyses) were concentrated to 25-30 mg/ml, then frozen in liquid nitrogen and stored at -80 °C.

Expression and Purification of His_6-DdSkp1

DNA encoding for *N*-terminally His_6-tagged *Dictyostelium discoideum* full-length DdSkp1 (aa 1-163, DdSkp1) was cloned into the pNIC28-Bsa4 vector (88). Recombinant DdSkp1 protein was produced in *Escherichia coli* BL21 GOLD (DE3) cells. The cells were grown at 37 °C in 2TY media supplemented with 10 μg/ml kanamycin to an OD_{600} = 1.2. Expression was initiated by 0.2 mM IPTG. Growth was continued at 28 °C for another 16-18 hours; cells were harvested by centrifugation (10,000g, 8 minutes) and stored at -80 °C. The cell pellets were resuspended in lysis buffer (50 mM HEPES pH 7.6, 500 mM NaCl, 5 mM imidazole, and DNaseI (bovine pancreas,

grade II, Roche)). Cells were lysed by sonication on ice (Sonics Vibracell VCX750 Ultrasonic Cell Disruptor) 60 % amplitude, 10 cycles of 30 s sonication with 40 s rest between each cycle. The lysates were filtered using a Minisart 0.45 µm syringe filter (Sartorius Stedim Biotech) then loaded onto a 5 ml HisTrap column (GE Healthcare), that had been equilibrated with binding buffer (50 mM HEPES pH 7.6, 500 mM NaCl, 5 mM imidazole) and separated using an AKTA purifier (GE Healthcare) carried out at 4 °C with a flow rate of 1 ml/min. The column was washed with wash buffer (50 mM HEPES pH 7.6, 500 mM NaCl, 30 mM imidazole). His_6-DdSkp1 was eluted with elution buffer (50 mM HEPES pH 7.6, 500 mM NaCl, 500 mM imidazole) using a linear gradient (0-100 %). Based on the UV-trace (280 nm) and SDS-PAGE, fractions containing purified His_6-DdSkp1 were pooled. The combined fractions were then treated with the TEV protease (1 mg / 50 mg target protein) and 50 mM EDTA overnight at 4 °C to cleave the His_6 tag and remove metal ions. The protein solution was then concentrated to 2.5 ml using a 10 kDa MW cut-off 15 ml concentrator (Amicon®). The concentrated protein was exchanged into binding buffer (50 mM HEPES pH 7.6, 500 mM NaCl, 5 mM imidazole) using a PD-10 desalting column (GE Healthcare). To remove the TEV protease, the solution was then loaded onto a 5 ml HisTrap column (GE Healthcare). The flow-through containing DdSkp1 was collected and concentrated using a 10 kDa MW cut-off 15 ml concentrator (Amicon®), then loaded onto a Superdex 75 size-exclusion column (GE Healthcare), which had been pre-equilibrated with size-exclusion buffer (50 mM HEPES pH 7.6), for further purification. The desired fractions (based on UV-trace and SDS-PAGE analyses) were concentrated to 25-30 mg/ml, then frozen in liquid nitrogen and stored at -80 °C.

Expression and Purification of His_6-TgSkp1

DNA encoding for *N*-terminally His_6-tagged *Toxoplasma gondii* full-length His_6-TgSkp1 (aa 1-170, TgSkp1) was cloned into the pNIC28-Bsa4 vector (88). Recombinant TgSkp1 protein was produced in *Escherichia coli* BL21 GOLD (DE3) cells. The cells were grown at 37 °C in 2TY media supplemented with 10 µg/ml kanamycin to an OD_{600} = 1.2. Expression was initiated by 0.2 mM IPTG. Growth was continued at 28 °C for another 16-18 hours; cells were harvested by centrifugation (10,000g, 8 minutes) and stored at -80 °C. Cell pellets were resuspended in lysis buffer (50 mM HEPES pH 7.6, 500 mM NaCl, 5 mM imidazole, and DNaseI (bovine pancreas, grade II, Roche)). Cells were lysed by sonication on ice (Sonics Vibracell VCX750 Ultrasonic Cell Disruptor) 60 % amplitude, 10 cycles of 30 s sonication with 40 s rest between each cycle. The lysates were filtered using a Minisart 0.45 µm syringe filter (Sartorius Stedim Biotech) then loaded onto a 5 ml HisTrap column (GE Healthcare), that had been equilibrated with binding buffer (50 mM HEPES pH 7.6, 500 mM NaCl, 5 mM imidazole) and separated using an AKTA purifier (GE Healthcare) carried out at 4 °C with a flow rate of 1 ml/min. The column was washed with wash buffer (50 mM HEPES pH 7.6, 500 mM NaCl, 30 mM imidazole). His_6-TgSkp1 was eluted with elution buffer (50 mM HEPES pH 7.6, 500 mM NaCl, 500 mM imidazole) using a linear gradient (0-100 %). Based on the UV-trace (280 nm) and SDS-PAGE, fractions containing purified His_6-TgSkp1 were pooled. The combined fractions were then treated with the TEV protease (1 mg / 50 mg target protein) and 50 mM EDTA overnight at 4 °C to cleave the His_6 tag and remove metal ions. The protein solution was then concentrated to 2.5 ml using a 10 kDa MW cut-off 15 ml concentrator (Amicon®). The concentrated protein was exchanged into binding buffer (50 mM HEPES pH 7.6, 500 mM NaCl, 5 mM imidazole) using a PD-10 desalting column (GE Healthcare). To remove the TEV protease, the solution was then loaded onto a 5 ml HisTrap column

(GE Healthcare). The flow-through containing TgSkp1 was collected and concentrated using a 10 kDa MW cut-off 15 ml concentrator (Amicon®), then loaded onto a Superdex 75 size-exclusion column (GE Healthcare), which had been pre-equilibrated with size-exclusion buffer (50 mM HEPES pH 7.6), for further purification. The desired fractions (based on UV-trace and SDS-PAGE analyses) were re-concentrated to 25-30 mg/ml, then frozen in liquid nitrogen and stored at -80 °C.

Expression and Purification of HsSkp1-His$_6$ and HsSkp1-His$_6$ E147P

DNA encoding for C-terminally His6-tagged Homo sapiens full-length HsSkp1 (aa 1-163, HsSkp1) was cloned into the pET-21a(+) vector. Site-directed mutagenesis was conducted using a Q5® Site Directed Mutagenesis Kit (New England Biolabs) to mutate Glu147 of HsSkp1 to proline, HsSkp1 E147P. The primers designed specifically for mutating E147P were: Forward, 5'–CAATATCAAAAATGACTTTACTCCGGAGGAGGAAGCCCAGGTACGC–3'; Reverse, 5'–GCGTACCTGGGCTTCCTCCTCCGGAGTAAAGTCATTTTTGATATTG–3'. The DNA was amplified by the polymerase chain reaction (PCR) using a thermal cycler. HsSkp1-His$_6$ and HsSkp1-His$_6$ E147P proteins were independently produced using the same methods in *Escherichia coli* BL21 GOLD (DE3) cells. The cells were grown at 37 °C in 2TY media supplemented with 10 µg/ml kanamycin to an OD_{600} = 1.2. Expression was initiated by addition of 0.1 mM IPTG. Growth was continued at 28 °C for another 16-18 hours; cells were harvested by centrifugation (10,000g, 8 minutes) and stored at -80 °C. The cell pellets were resuspended in lysis buffer (50 mM HEPES pH 7.6, 500 mM NaCl, 5 mM imidazole, and DNaseI (bovine pancreas, grade II, Roche)). Cells were lysed by sonication on ice (Sonics Vibracell VCX750 Ultrasonic Cell Disruptor) 60 % amplitude, 10 cycles of 30 s sonication with 40 s rest between each cycle. The lysates were filtered using a Minisart 0.45 µm syringe filter (Sartorius Stedim Biotech) then loaded onto a 5 ml HisTrap column (GE Healthcare), that had been equilibrated with binding buffer (50 mM HEPES pH 7.6, 500 mM NaCl, 5 mM imidazole) and separated using an AKTA purifier (GE Healthcare) carried out at 4 °C with a flow rate of 1 ml/min. The column was washed with wash buffer (50 mM HEPES pH 7.6, 500 mM NaCl, 30 mM imidazole). HsSkp1-His$_6$ was eluted with elution buffer (50 mM HEPES pH 7.6, 500 mM NaCl, 500 mM imidazole) using a linear gradient (0-100 %). Based on the UV-trace (280 nm) and SDS-PAGE, fractions containing purified HsSkp1-His$_6$ or HsSkp1-His$_6$ E147P were pooled. The protein solution was then concentrated to 2.5 ml using a 10 kDa MW cut-off 15 ml concentrator (Amicon®). The concentrated protein was exchanged into storage buffer (100 mM HEPES pH 7.6) using a PD-10 desalting column (GE Healthcare). The flow-through containing HsSkp1-His$_6$ or HsSkp1-His$_6$ E147P was collected and re-concentrated using a 10 kDa MW cut-off 15 ml concentrator (Amicon®) to 25-30 mg/ml, then frozen in liquid nitrogen and stored at -80 °C.

Expression and Purification of HsPHD2

DNA encoding for *Homo sapiens* HsPHD2 (aa 181-426) was cloned into the pET-24a(+) vector. HsPHD2 was produced in *Escherichia coli* BL21 GOLD (DE3) cells. The cells were grown at 37 °C in 2TY media supplemented with 10 µg/ml kanamycin to an OD_{600} = 1.2. Expression was initiated by 0.5 mM IPTG. Growth was continued at 28 °C for another 16-18 hours; cells were harvested by centrifugation (10,000g, 8 minutes) and stored at -80 °C. The cell pellets were

resuspended in lysis buffer (50 mM HEPES pH 7.6, 500 mM NaCl, 5 mM imidazole, and DNaseI (bovine pancreas, grade II, Roche)). Cells were lysed by sonication on ice (Sonics Vibracell VCX750 Ultrasonic Cell Disruptor) 60 % amplitude, 10 cycles of 30 s sonication with 40 s rest between each cycle. The lysates were filtered using a Minisart 0.45 µm syringe filter (Sartorius Stedim Biotech) then loaded onto a 50 ml SP Sepharose® Fast Flow column (GE Healthcare) with a flow rate of 1 ml/min, that had been equilibrated with Buffer A (100 mM MES hydrate, pH 5.8) and separated using an AKTA purifier (GE Healthcare) carried out at 4 °C with a flow rate of 1 ml/min. HsPHD2 was eluted with Buffer B (100 mM MES hydrate, 2 M NaCl, pH 5.8). Based on the UV-trace (280 nm) and SDS-PAGE, fractions containing purified HsPHD2 were pooled. The protein solution was then concentrated to 2.5 ml using 30 kDa molecular weight cut-off 15 ml concentrator (Amicon®). The concentrated protein loaded onto a Superdex 75 size-exclusion column (GE Healthcare), which had been pre-equilibrated with degassed size-exclusion buffer (100 mM HEPES, 150 mM NaCl, pH 7.5), for further purification. The desired fractions (based on UV-trace and SDS-PAGE analyses) were concentrated to 40-50 mg/ml, then frozen in liquid nitrogen and stored at -80 °C.

Kinetic Assay Conditions

DdPhyA kinetic assays

Assay conditions for DdPhyA varying full-length DdSkp1 substrate concentration:

DdPhyA (1 µM), full-length DdSkp1 (50 µM, 100 µM, 200 µM, 300 µM, 400 µM, 600 µM, 800 µM, or 1000 µM), $(NH_4)_2Fe(II)(SO_4)_2$ (50 µM), sodium L-ascorbate (1 mM) and 2-oxoglutarate disodium salt (500 µM) in HEPES (100 mM) pH 7.6, at 37 °C for 4 minutes. Incubations were quenched with 1 % (*v/v*) aqueous formic acid, then analyzed using LC-ESI-MS (Xevo G2-S QToF mass spectrometer equipped with an electrospray ionization source (Waters®) coupled with a Waters® ACQUITY UPLC System).

Assay conditions for DdPhyA varying 2OG concentration:

DdPhyA (1 µM), full-length DdSkp1 (100 µM), $(NH_4)_2Fe(II)(SO_4)_2$ (50 µM), sodium L-ascorbate (1 mM) and 2-oxoglutarate disodium salt (0 µM, 20 µM, 50 µM, 100 µM, 200 µM, and 300 µM) in HEPES (100 mM) pH 7.6. The reactions were incubated at 37 °C for 6 minutes before being quenched with 1 % (*v/v*) aqueous formic acid, then analyzed using solid phase extraction (SPE) coupled to MS (Xevo G2-S Q-tof mass spectrometer equipped with an electrospray ionization source (Waters®) coupled with a Waters® ACQUITY UPLC System).

Assay conditions for DdPhyA varying O_2 concentration:

DdPhyA (1 µM), full-length DdSkp1 (600 µM), $(NH_4)_2Fe(II)(SO_4)_2$ (50 µM), sodium L-ascorbate (1 mM) and 2-oxoglutarate disodium salt (500 µM) in HEPES (100 mM) pH 7.6, under O_2 5%, 10%, 15%, 20%, 30%, 40%, 50%, and 60%. Reactions were incubated at 37 °C for 8.25 minutes before being quenched with 1 % (*v/v*) aqueous formic acid, then analyzed using LC-ESI-MS as above.

TgPhyA kinetics

Assay conditions for TgPhyA varying the full-length TgSkp1 substrate concentration:

TgPhyA (1 µM), full-length TgSkp1 substrate (100 µM, 200 µM, 300 µM, 400 µM, 600 µM, 800 µM, 1000 µM, and 1200 µM), $(NH_4)_2Fe(II)(SO_4)_2$ (50 µM), sodium L-ascorbate (1 mM) and 2-oxoglutarate disodium salt (500 µM) in HEPES (100 mM) pH 7.6. Reactions were incubated at 37 °C for 2 minutes before being quenched with 1 % (*v/v*) aqueous formic acid, then analyzed using LC-ESI-MS as above.

Assay conditions for TgPhyA varying 2OG concentration:

TgPhyA (1 µM), full-length TgSkp1 (600 µM), $(NH_4)_2Fe(II)(SO_4)_2$ (50 µM), sodium L-ascorbate (1 mM) and 2-oxoglutarate disodium salt (0 µM, 20 µM, 50 µM, 100 µM, 200 µM, 300 µM, 400 µM, 600 µM, and 800 µM) in HEPES (100 mM) pH 7.6. The reactions were incubated at 37 °C for 6 minutes before being quenched with 1 % (*v/v*) aqueous formic acid, then analyzed using solid phase extraction (SPE) coupled to MS (Xevo G2-S Q-tof mass spectrometer equipped with an electrospray ionization source (Waters®) coupled with a Waters® ACQUITY UPLC System).

Assay conditions for TgPhyA varying the O_2 concentration:

TgPhyA (1 µM), full-length TgSkp1 substrate (600 µM), $(NH_4)_2Fe(II)(SO_4)_2$ (50 µM), sodium L-ascorbate (1 mM) and 2-oxoglutarate disodium salt (500 µM) in HEPES (100 mM) pH 7.6, under O_2 1%, 2%, 5%, 10%, 15%, 20%, and 30%. Reactions were incubated at 37 °C for 2 minutes, then quenched with 1 % (*v/v*) aqueous formic acid, then analyzed using SPE coupled with MS as above.

Table S1. Peptides used in this study.

Oligopeptides were synthesized using a Liberty Blue™ Automated Microwave Peptide Synthesizer (CEM Corporation) with C-terminal amides. Except, for the NMR studies (Figure S5), all DdSkp1, TgSkp1, and HsSkp1 substrates used in this study were full-length proteins; their sequences are reported in Figure 1.

Peptide	Sequence
HIF-1α CTAD (789-822)	DESGLPQLTSYDCEVNAPIQGSRNLLQGEELLRAL
HIF-2α CTAD (832-866)	ESYLLPELTRYDCEVNVPVLGSSTLLQGGDLLRAL
HIF-1α CODD (556-574)	DLDLEMLAPYIPMDDDFQL
HIF-1α NODD (395-413)	DALTLLAPAAGDTIISLDF
HIF-2α CODD (523-541)	ELDLETLAPYIPMDGEDFQ
HIF-2α NODD (398-416)	EELAQLAPTPGDAIISLDF

Figures and Tables

Figure S1. DdPhyA, PPHD (PDB 4J25), TgPhyA (Gene ID: TGGT1_232960), and TgPhyB (GenBank: KYF42913.1) sequences.

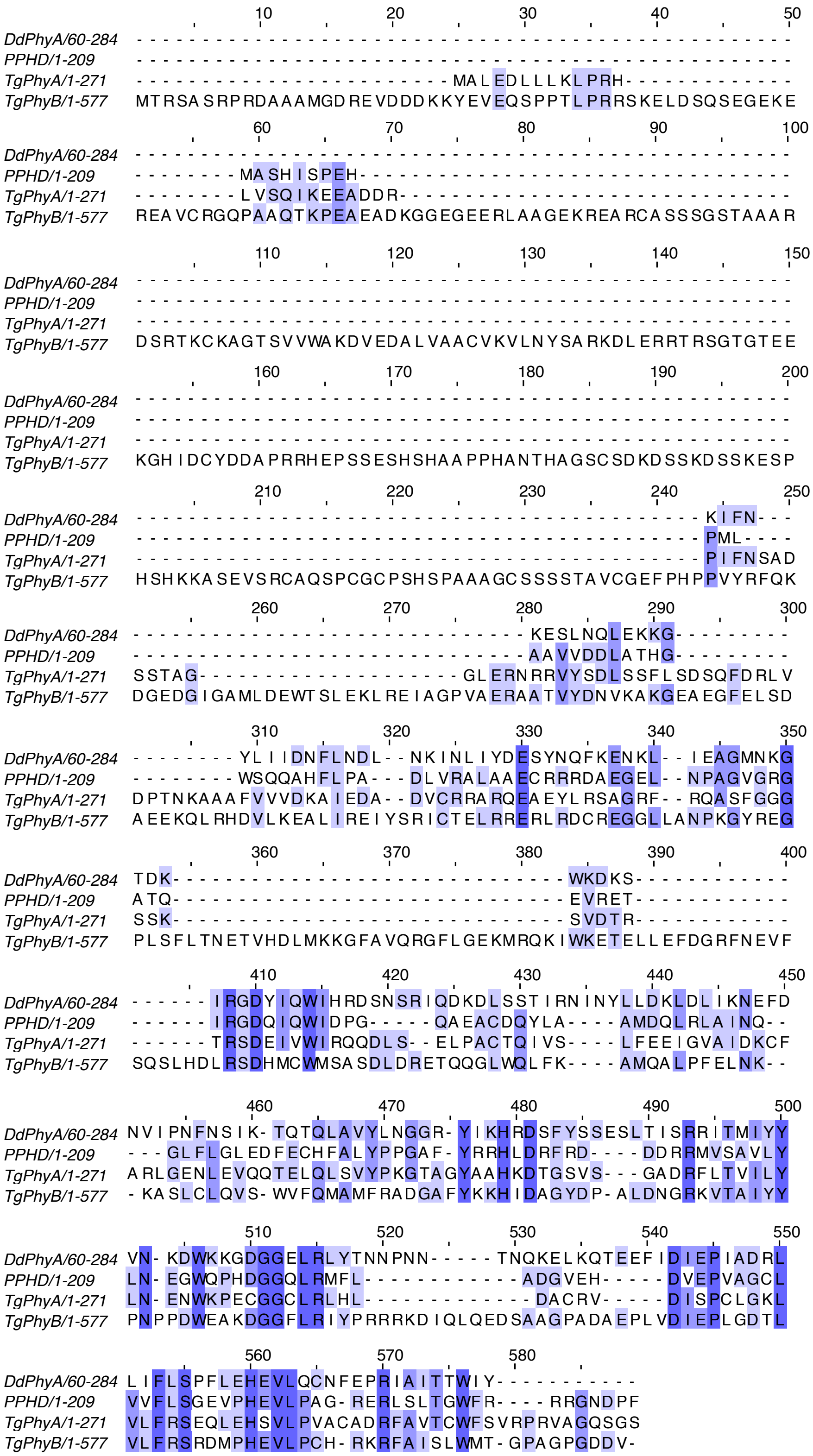

Figure S2. Sequence alignment of peptide sequences obtained by Nano LC-MS/MS with DdSkp1 and TgSkp1 sequence.

(5) (A) Parts of the trypsin digested peptide sequences aligned with the DdSkp1 sequence from the hydroxylated sample containing full-length DdSkp1 protein, DdPhyA, Fe(II), and 2OG. The results imply proline 143 is hydroxylated. **(B)** No enzyme control peptide sequences aligned with the DdSkp1 sequence from the sample containing full-length DdSkp1 protein. The results imply proline 143 is not hydroxylated. **(C)** Parts of trypsin digested peptide sequences aligned with TgSkp1 sequence from the hydroxylated sample containing TgPhyA, Fe(II), and 2OG. The results imply proline 154 is hydroxylated. **(D)** No enzyme control peptide sequences aligned with TgSkp1 sequence from the sample containing full-length TgSkp1 protein. The results imply proline 154 is not hydroxylated. **(E)** Potential modifications as predicted by PEAKS® 8.0 (Bioinformatics Solutions Inc., Waterloo, Canada) (6). Peptides were analyzed using a NanoACQUITY-UPLC system (Waters®) machine coupled to an Orbitrap Elite™ mass spectrometer (Thermo Fisher Scientific™) possessing an EASY-Spray nano-electrospray ion source (Thermo Fisher Scientific™).

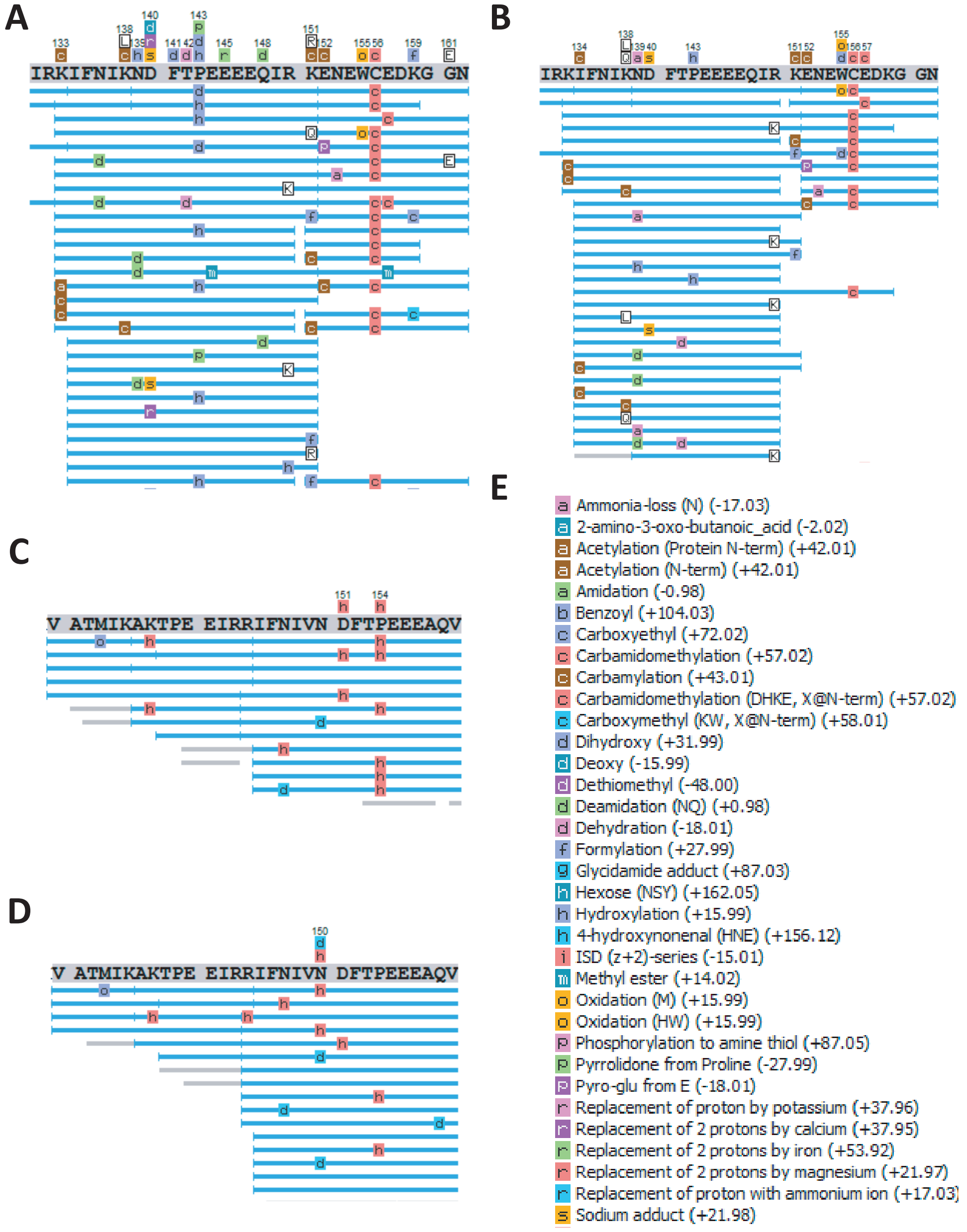

Figure S3. Assignment of modification sites of DdSkp1 by DdPhyA as determined by LC coupled to tandem MS (nano MS/MS).

(A) MS/MS analysis of the peptide containing proline 143 generated by in-solution trypsin digestion of DdSkp1 after incubation with Fe(II), 2OG, and ascorbate (37 °C, 1 hour) without DdPhyA. **(B)** MS/MS analysis of the peptide containing the hydroxylated proline 143 generated by in-solution trypsin digestion of DdSkp1 protein after incubation with DdPhyA and cofactors/cosubstrates. Assays contained: DdPhyA (1 µM), full-length DdSkp1 protein substrate (100 µM), $(NH_4)_2Fe(II)(SO_4)_2$ (50 µM), sodium L-ascorbate (1 mM), and 2-oxoglutarate disodium salt (500 µM) in HEPES (100 mM), pH 7.6. Reactions were incubated at 37 °C for 1 hour and quenched using an equal volume of 1% (v/v) aqueous formic acid, before trypsin digestion. In-solution digestion conditions: trypsin (20 ng/µl) in 50 mM NH_4HCO_3 for 30 minutes. Prior to digestion, samples were reduced (45 minutes, 10 mM DTT in 100 mM NH_4HCO_3) and alkylated (30 minutes, 55 mM of iodoacetamide in 100 mM NH_4HCO_3). In-gel digestion was carried out using the same procedure with the relevant excised band after SDS-PAGE gel separation.

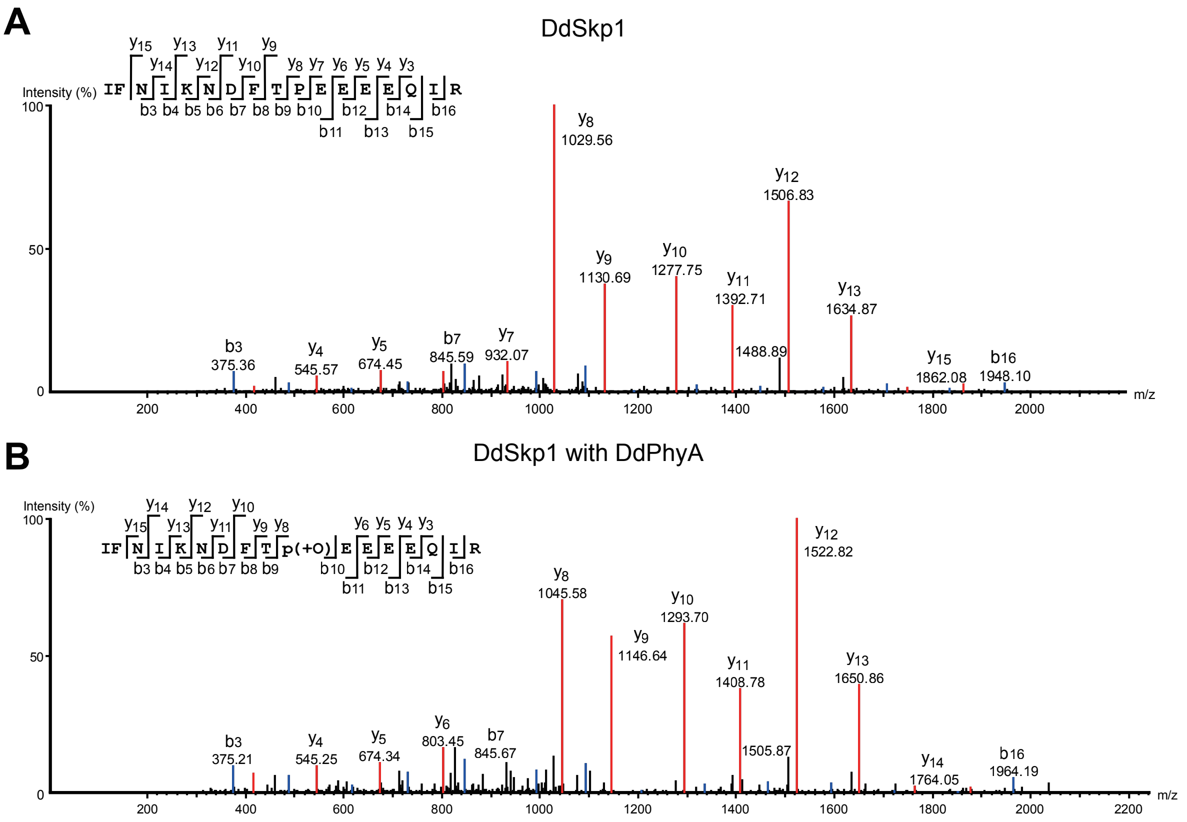

Figure S4. Assignment of modification sites of TgSkp1 by TgPhyA as determined by LC coupled to tandem MS (nano MS/MS).

(A) MS/MS analysis of the peptide containing proline 154 generated by in-solution trypsin digestion of TgSkp1 protein after incubation with Fe(II), 2OG, and ascorbate (37 °C, 1 hour) without TgPhyA. **(B)** MS/MS analysis of the peptide containing the hydroxylated proline 154 generated by in-gel trypsin digestion of TgSkp1 protein after incubation with TgPhyA and cofactors / cosubstrates. Assays contained TgPhyA (1 µM), full-length TgSkp1 protein substrate (100 µM), $(NH_4)_2Fe(II)(SO_4)_2$ (50 µM), sodium L-ascorbate (1 mM), and 2-oxoglutarate disodium salt (500 µM) in HEPES (100 mM), pH 7.6. Reactions were incubated at 37 °C for 1 hour, then quenched using an equal volume of 1% (v/v) aqueous formic acid, before trypsin digestion. In-solution digestion conditions: trypsin (20 ng/µl) in 50 mM NH_4HCO_3 for 30 minutes. Prior to digestion, samples were reduced (45 minutes, 10 mM DTT in 100 mM NH_4HCO_3) and alkylated (30 minutes, 55 mM of iodoacetamide in 100 mM NH_4HCO_3). In-gel digestion was carried out using the same procedure with the relevant excised cut band after SDS-PAGE gel separation.

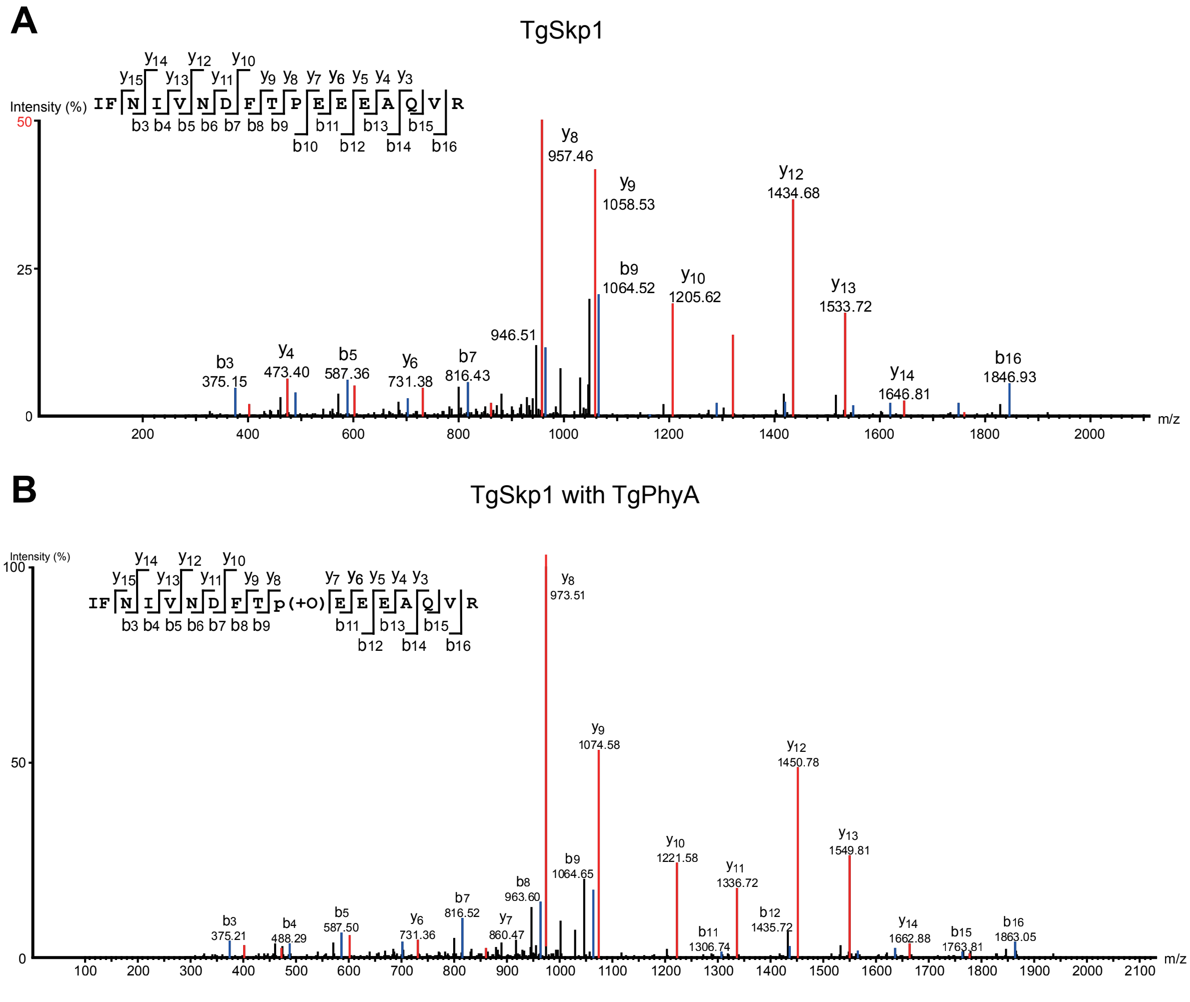

Figure S5. NMR analysis of the prolyl-hydroxylation regiochemistry of DdPhyA and TgPhyA. **(A)** 2D total correlation spectroscopy (TOCSY) spectrum (700 MHz) of hydroxylated DdSkp1 peptide (FNIKNDFTHy<u>P</u>EEEEQIRKENEWCEDK-NH_2) containing a 4-hydroxyprolyl residue. **(B)** 2D TOCSY spectrum (700 MHz) of TgSkp1 peptide (FNIVNDFTHy<u>P</u>EEEAQVREENK-NH_2) containing a 4-hydroxyprolyl residue. The δ- and β-signals corresponding to the protons of the hydroxylated prolyl residues are indicated with lines. The distinctive chemical shifts of δ- and β- protons are consistent with the presence of a 4-hydroxyprolyl residue in both hydroxylated DdSkp1 and TgSkp1. **(C)** Structure of a 4(γ)-hydroxylated prolyl residue with the carbon atoms labeled. **(D)** Table showing the average proton chemical shifts of unhydroxylated prolyl residues from the Biological Magnetic Resonance Data Bank as compared to the chemical shifts of the protons of 4-hydroxyprolyl residues of DdSkp1 and TgSkp1.

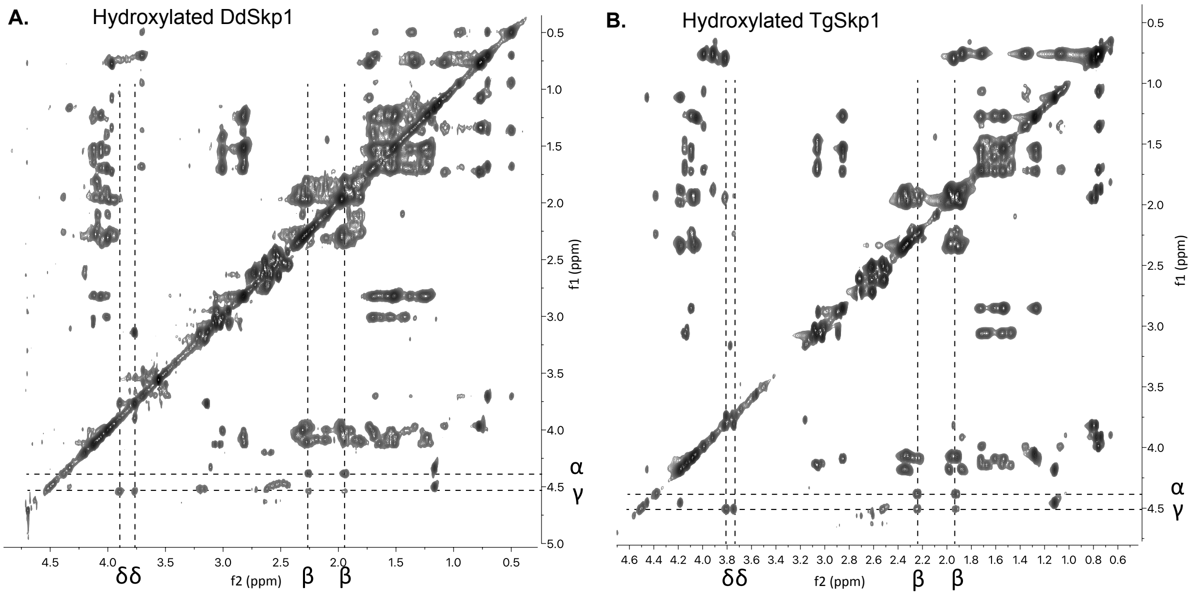

C.

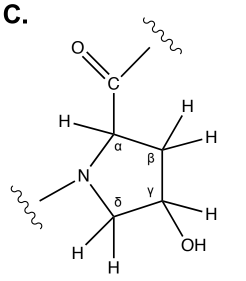

D.

Proline Chemical Shift

Position	Average Chemical Shift of Proline	Hydroxylated Proline of DdSkp1	Hydroxylated Proline of TgSkp1
H^{α}	4.39	4.0	4.3
$H^{\beta 2}$	2.08	1.9	1.9
$H^{\beta 3}$	2.00	2.3	2.2
$H^{\gamma 2}$	**1.92**	**4.5**	**4.5**
$H^{\gamma 3}$	**1.90**		
$H^{\delta 2}$	3.65	3.7	3.7
$H^{\delta 3}$	3.61	3.9	3.8

Figure S6. Analyses of 2OG turnover to succinate by ¹H NMR.

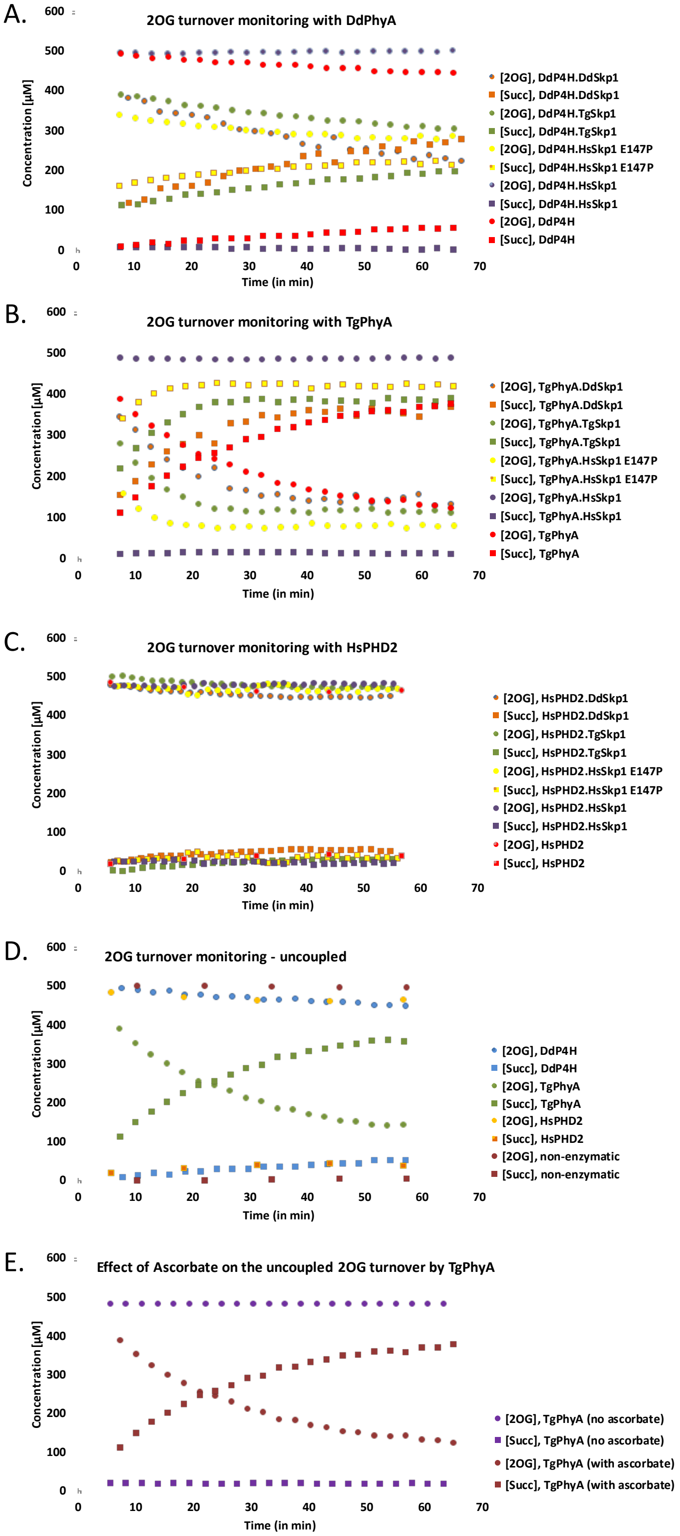

(A) Analyses of 2OG turnover to succinate by DdPhyA by ¹H NMR. 2OG concentrations: circles; succinate concentrations: squares. Time courses of DdPhyA with different substrates are distinguished by different colors, i.e. orange (DdSkp1), green (TgSkp1), yellow (HsSkp1 E147P), purple (HsSkp1), and red (no substrate). **(B) Analyses of 2OG turnover to succinate by TgPhyA by ¹H NMR.** Time course of TgPhyA with different substrates are distinguished by different colors, i.e. orange (DdSkp1), green (TgSkp1), yellow (HsSkp1 E147P), purple (HsSkp1) and red (no substrate). **(C) Analyses of 2OG turnover to succinate by PHD2 by ¹H NMR.** Time courses of PHD2 with different substrates are distinguished by different colors, i.e. orange (DdSkp1), green (TgSkp1), yellow (HsSkp1 E147P), and purple (HsSkp1). **(D, E) Analyses of 2OG turnover to succinate by ¹H NMR in the absence of substrate. (D)** Comparison of uncoupled 2OG turnover between HsPHD2 (orange), DdPhyA (blue), TgPhyA (green) and non-enzymatically (burgundy). **(E)** Substrate uncoupled 2OG turnover by TgPhyA with (burgundy) and without (purple) ascorbate.

Figure S7. ^{1}H NMR analyses of 2OG turnover to succinate.

(A) and **(B)** show differences in substrate 'uncoupled' 2OG reactions as catalyzed by DdPhyA and TgPhyA in the absence of substrate. TgPhyA catalyzes high levels of uncoupled turnover, in a manner highly dependent on ascorbate (Figure S6E). **(C)** The addition of HsSkp1 to TgPhyA, even in the presence of ascorbate (Table S2) reduces the extent of reaction of 2OG to give succinate. Thus HsSkp1, though not a TgPhyA substrate, binds to TgPhyA, in a manner modulating 2OG reaction.

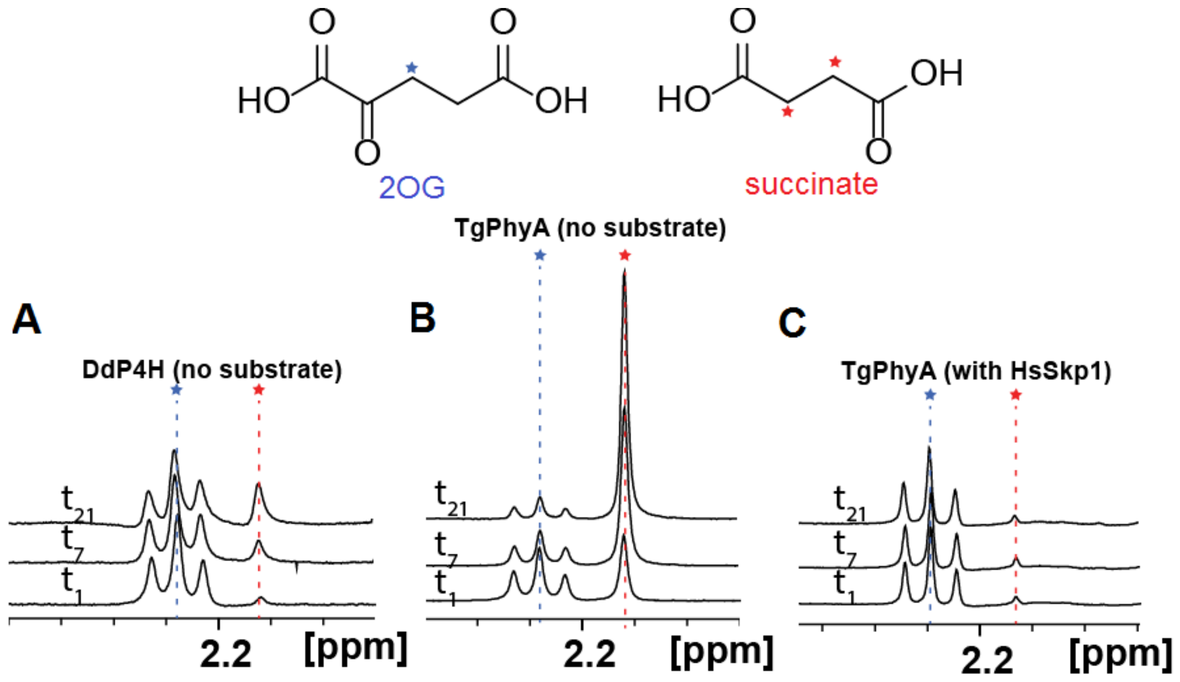

Table S2. 2OG turnover assay conditions.

Assay Conditions (Figures S6-7)	Final Concentration
Enzyme	20 µM
Substrate	400 µM
2-Oxoglutarate acid disodium salt (Sigma-Aldrich, K3752)	500 µM
Sodium L-ascorbate (Sigma-Aldrich, 11140)	1 mM (or 0 mM, see Figure S6E)
Ammonium iron(II) sulfate hexahydrate (Sigma-Aldrich, 215406)	50 µM

Figure S8. ^{1}H-edited CPMG studies on the proposed binding of 2OG to Skp1.

2OG was not observed to bind to any of the Skp1 proteins under the tested conditions. Assay mixtures: 1:1; 2OG:Skp1 (50 µM) buffered in 50 mM Tris-D_{11} pH 7.5 in 9:1; H_2O:D_2O.

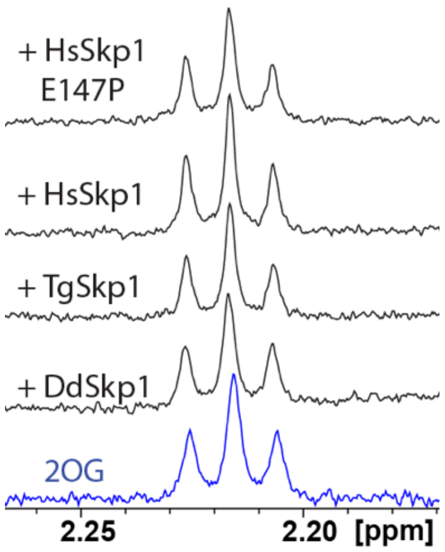

Figure S9. T_m shift assays with (A) DdPhyA or (B) TgPhyA.

Results shown are the mean ±s.d. of the T_m in triplicate using *apo*-DdPhyA or *apo*-TgPhyA as reference. Assay conditions: *apo*-DdPhyA or *apo*-TgPhyA (4 µM), metal chloride salt (50 µM), 2OG or NOG disodium salt (50 µM), and SYPRO® Orange (Invitrogen) in HEPES (100 mM) pH 7.6. Differential Scanning Fluorimetry was used to determine T_m values using C1000 Touch™ Thermal Cycler (BioRad). Data were analyzed using GraphPad Prism. *Apo* refers to EDTA-treated, non-metallated enzyme.

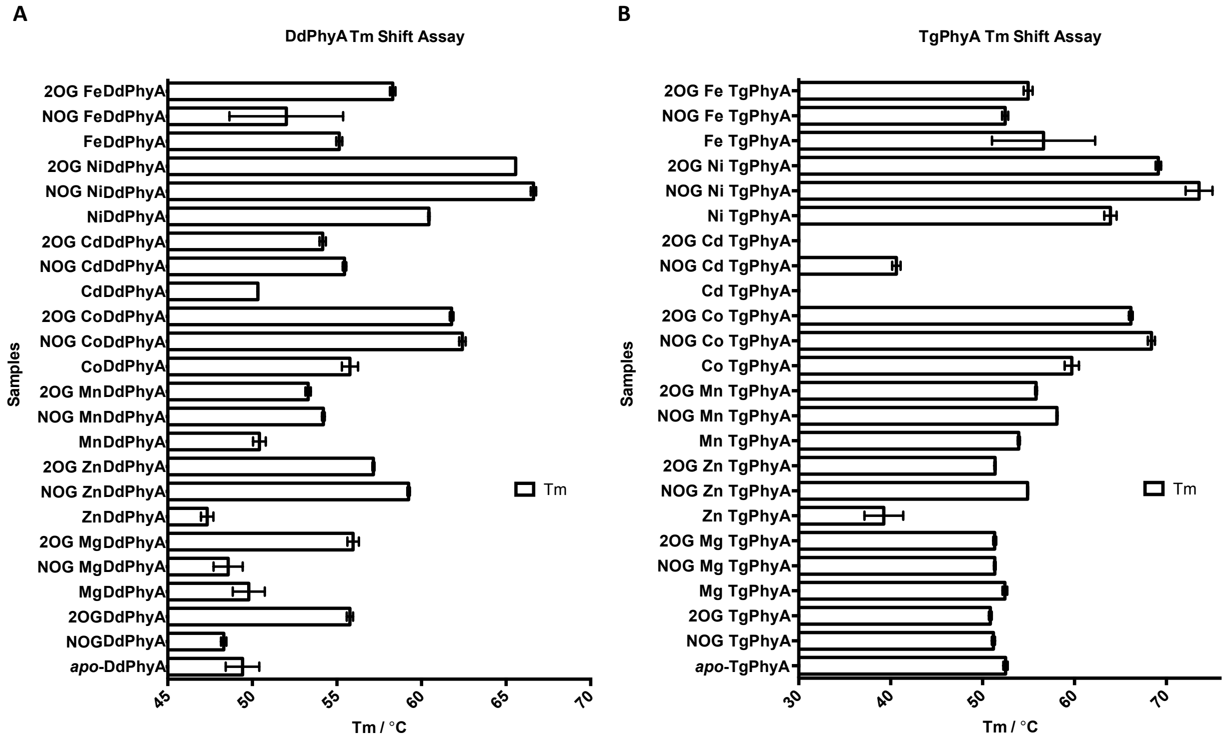

Table S3. Assay conditions for the T_m shift assays with DdPhyA and TgPhyA.

Assay Reagent	Final Conc. µM	Stock Conc. µM	Volume Per Assay µl
2OG or NOG	50	500	5
Enzyme	4	20	10
Metal	50	1000	2.5
100 mM HEPES pH 7.6	1:2.5 SYPRO Orange: Buffer		32.5
Total volume	-		50

Figure S10. Comparison of DdPhyA structure with those of other prolyl hydroxylases (complexed with substrates).

(A,B) Comparison of DdPhyA.Ni.NOG with PPHD.Mn.NOG (PDB ID: 4J25) and PPHD.Mn.NOG.EF-Tu (PDB ID: 4IW3). **(C,D)** Superimposition of the DdPhyA.Ni.NOG structure with that of TaPHD.Mn.NOG.TaODD (PDB ID: 6F0W). **(E,F)** Superimposition of the DdPhyA.Ni.NOG structure with that of *Chlamydomonas reinhardtii* CrP4H.Zn.AcOH.(SerPro)$_5$ (PDB: 3GZE). Note, for each alignment in the figure the substrate is present is for the non-DdPhyA structure, but not for DdPhyA.

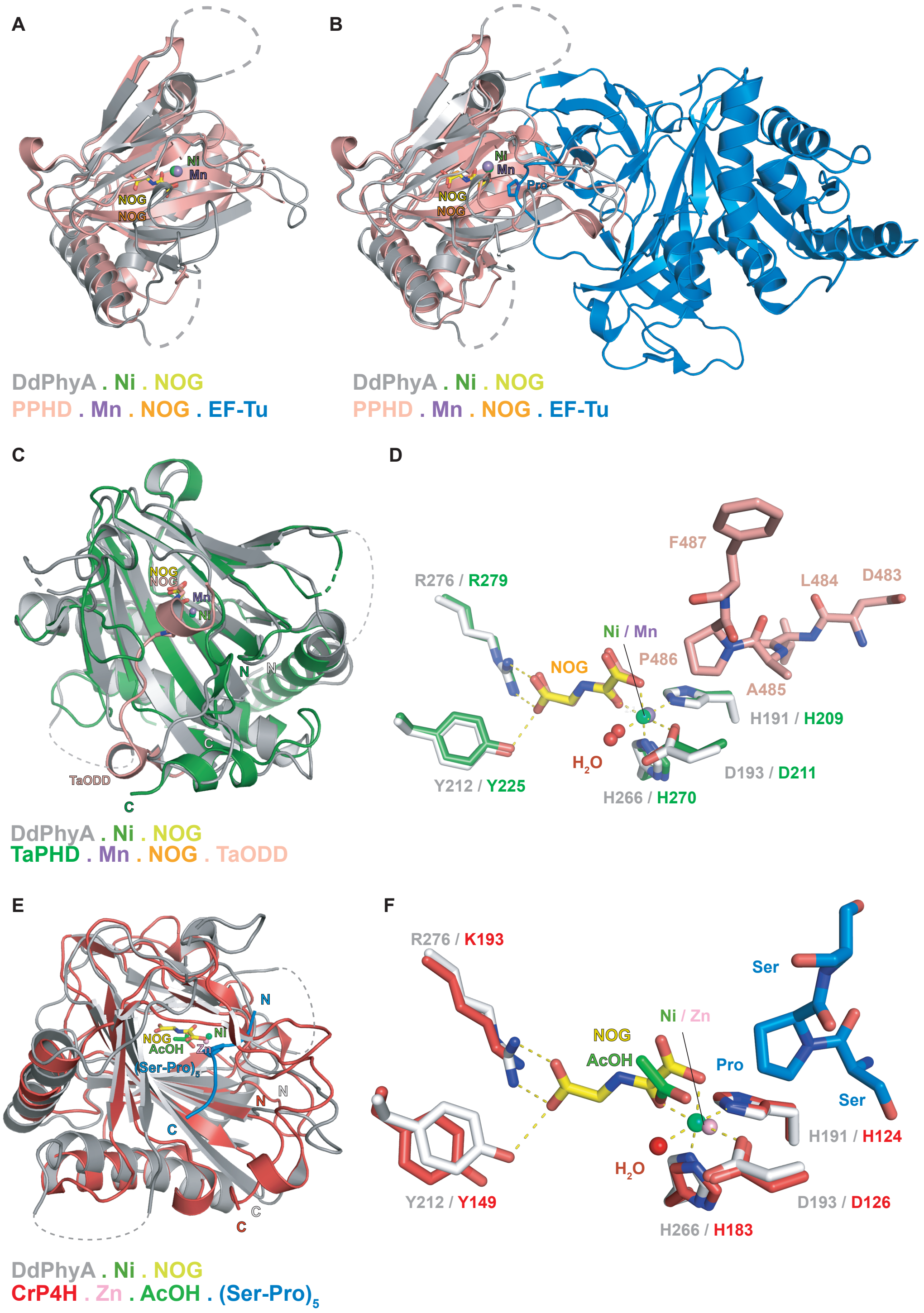

Figure S11. Comparison of DdPhyA and Pseudomonas putida PPHD structures.

(A) Superimposition of structures for the DdPhyA.Ni.NOG and the *P. putida* PPHD:EF-Tu (PDB ID: 4IW3) complexes. **(B)** Superimposition of the active site views of the two enzymes. EF-Tu is bound to PPHD, no substrate is present for DdPhyA. **(C)** Sequence alignment of DdPhyA (60-284) and PPHD. Secondary structure elements labelled and indicated as helix (spirals) and strand (arrows) above (DdPhyA, gray) and below (PPHD, pink) aligned sequence. Residues involved in active site Fe(II) and 2OG binding are indicated in yellow text.

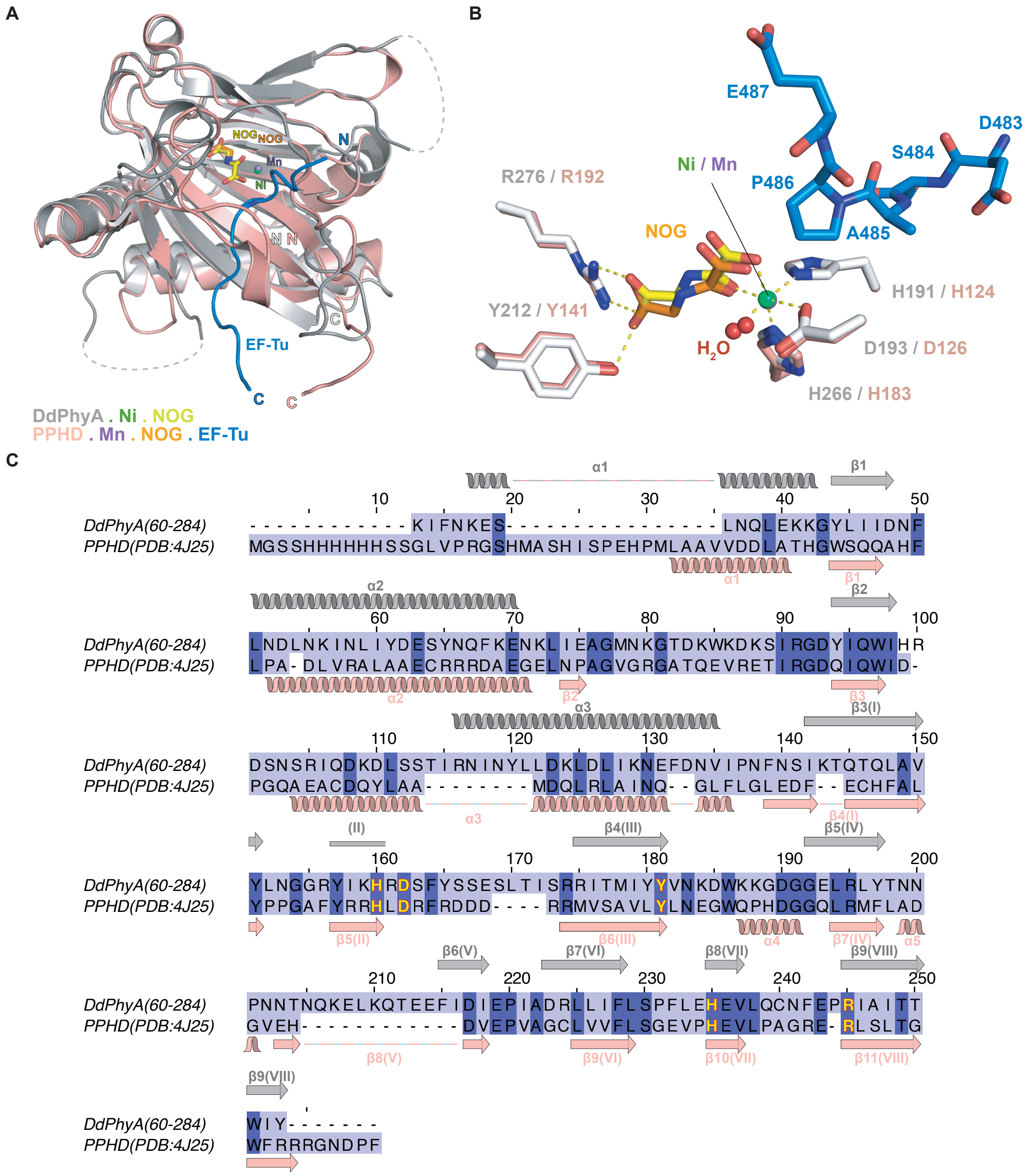

Supplement: Supplementary file 1 [file mmc1.pdf]
